# Integrative Phytochemical, Antioxidant, and NLRP3‐Linked and Anti‐Inflammatory Evaluation of *Salvia balansae* (Aurès Chemotype): Insights Into Its Safety and Therapeutic Potential

**DOI:** 10.1002/open.70280

**Published:** 2026-08-20

**Authors:** Khadra Afaf Bendrihem, Messaoud Hachemi, Mohamed Aimene Mihoubi, Fares Mohamed Amine, Majid Sharifi‐Rad, Dalal Houhou, Rahma Guemmaz, Khaled Kherraz, Azzeddine Zeraib, Assia Abdelmalek, Aicha Mouane, Nour Elhouda Ayeb, Chawki Bensuici, Maria Chikha, Kamel Nagaz, Ayomide Victor Atoki, Mohammed Messaoudi

**Affiliations:** ^1^ Biotechnology, Water, Environment and Health Laboratory Faculty of Natural and Life Sciences University of Abbes Laghrour Khenchela Algeria; ^2^ Laboratory of Biotechnology of Bioactive Molecules and Cellular Physiopathology (LBMBPC) Faculty of Science and Nature's Life University of Batna 2 Batna Algeria; ^3^ Lab, Higher School of Saharan Agriculture – El Oued El Oued Algeria; ^4^ Laboratory of Science and Techniques for Living Institute of Agricultural and Veterinary Sciences Mohamed Cherif Messaadia University Souk Ahra Algeria; ^5^ Department of Range and Watershed Management Faculty of Water and Soil University of Zabol Zabol Iran; ^6^ Laboratory of Research on Bioactive Products and Biomass Valorization Department of Chemistry Higher Normal School of Kouba (ENS) Alger Algeria; ^7^ Laboratory of Genetics, Biotechnology and Valorization of Bio‐Resources University of Biskra Biskra Algeria; ^8^ Laboratory of Applied Biochemistry Department of Biotechnology Faculty of Natural and Life Sciences University Ferhat Abbas Setif‐1 Algeria; ^9^ Faculty of Life and Natural Sciences Department of Biotechnology Setif University Setif Algeria; ^10^ Biotechnology Research Center Ali Mendjeli UV 03 Constantine Algeria; ^11^ Drylands and Oases Cropping Laboratory IRA Medenine Medenine Tunisia; ^12^ Department of Biochemistry Kampala International University Ishaka Uganda

**Keywords:** antioxidant activity, croton oil ear edema, NLRP3‐linked anti‐inflammatory activity, phytochemical profiling, *Salvia balansae*, toxicological evaluation

## Abstract

*Salvia balansae*, an endemic Algerian species, remains poorly investigated despite the recognized medicinal importance of the *Salvia* genus. This study comprehensively evaluated its phytochemical composition, biological activities, and safety profile. Quantitative analyses revealed high levels of total phenolics, flavonoids, flavonols, condensed tannins, and triterpenoids, while LC–MS identified a distinctive Aurès‐region chemotype enriched in luteolin, rosmarinic acid, and flavonoid glycosides. The extract demonstrated strong antioxidant activity in free radical‐scavenging, metal‐chelating, and reducing‐power assays. Anti‐inflammatory activity was confirmed by inhibition of protein denaturation in vitro and significant suppression of croton oil‐induced ear edema and histopathological damage in vivo. Molecular docking predicted favorable binding of salvianolic acid, rutin, and apigenin‐7‐O‐glucoside to NLRP3 inflammasome components, suggesting a potential mechanism underlying the observed anti‐inflammatory effects. The extract also exhibited moderate inhibitory activity against cholinesterases and α‐amylase, indicating possible neuroprotective and antidiabetic potential. Acute oral toxicity testing at 2000 mg/kg showed no mortality, major clinical signs of toxicity, or apparent organ damage, although repeated‐dose studies are warranted. Collectively, these findings identify *S. balansae* as a polyphenol‐ and triterpenoid‐rich species with considerable promise for pharmaceutical and nutraceutical applications.

## Graphical abstract

An endemic Aurès chemotype of *Salvia balansae* emerges as a rich source of luteolin, rosmarinic acid, and other polyphenols with potent antioxidant and NLRP3‐linked anti‐inflammatory activities. Protection against experimental inflammation, favorable enzyme‐modulating properties, and a wide safety margin highlight its promise as a pharmaceutical and nutraceutical resource.

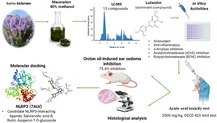

## 1 Introduction

Medicinal plants of the genus *Salvia* (Lamiaceae) represent one of the most diverse botanical groups, encompassing more than 900 species distributed across temperate and subtropical regions. Numerous members of this genus are well recognized in both traditional medicine and modern phytotherapy, largely due to their rich reservoir of bioactive metabolites, including polyphenols, terpenoids, and alkaloids, which exhibit antioxidant, anti‐inflammatory, antidiabetic, antimicrobial, and neuroprotective activities [1, 2]. Within this genus, *Salvia balansae* de Noé, an Algerian endemic species, remains one of the least explored taxa despite its ethnopharmacological importance in local healing practices [3].

Remarkably, *S. balansae* populations occur in two geographically isolated regions of Algeria: the coastal lowlands near Mostaganem and the high‐altitude Aurès Mountains situated over 500 km inland [4, 5]. Despite sharing a semiarid bioclimate, these populations exhibit subtle morphological differences, notably in leaf shape and flower coloration, likely reflecting local adaptation. The Aurès population, confined to the Oued Abdi valley, is considered a narrow endemic and remains largely uncharacterized in both phytochemical and biological terms [5, 6, 7]. This ecological and geographic distinctiveness underscores the originality and scientific relevance of investigating the Aurès chemotype of *S. balansae*.

Recent studies on *Salvia* species have highlighted the pharmacological potential of phenolic acids such as rosmarinic and caffeic acids, flavonoids including luteolin, apigenin, and quercetin derivatives, as well as pentacyclic triterpenoids like ursolic and oleanolic acids [8]. These metabolites are strongly implicated in modulating redox homeostasis, inflammatory signaling pathways, and enzyme activities relevant to metabolic and neurodegenerative disorders [9, 10]. Although several investigations have examined *S. balansae* from the coastal and low‐altitude regions of Algeria, particularly the Mostaganem population [4, 11, 12, 13], their findings cannot be generalized to all geographical variants of the species. To date, only the work of Bendrihem et al. [14] has focused on the Aurès population, providing preliminary insights into its composition. However, the phytochemical profile, biological properties, and chemotypic distinctiveness of this high‐altitude Aurès lineage remain largely unexplored, despite its morphological differentiation and unique environmental context, which suggest the possible existence of a distinct chemotype.

Inflammation and oxidative stress remain key contributors to chronic diseases such as diabetes, cancer, and neurodegeneration. The search for plant‐derived compounds capable of attenuating these processes is therefore of considerable biomedical and nutraceutical interest [15]. In this context, *S. balansae* provides an underexplored yet promising source of natural antioxidants and anti‐inflammatory agents. A comprehensive chemical characterization coupled with systematic biological evaluation is essential to elucidate its therapeutic potential and place it within the broader framework of *Salvia* phytomedicine.

Accordingly, the present study was designed to provide the first integrative phytochemical and biological characterization of the Aurès chemotype of *S. balansae*. We combined LC–MS‐based metabolite profiling with in vitro antioxidant and anti‐inflammatory assays, in vivo validation using croton oil‐induced ear edema in rats, and in silico molecular docking targeting the NLRP3 inflammasome. Furthermore, acute toxicity testing was performed to assess the safety margin of the extract. This integrative approach aims to elucidate the bioactive compounds in *S. balansae* and their potential roles in modulating oxidative stress and inflammation, thereby contributing to the growing evidence supporting the therapeutic value of North African endemic *Salvia* species.

## 2 Materials and Methods

### 2.1 Chemicals and Reagents

All solvents used (methanol, ethanol, acetone, and chloroform) were of analytical or HPLC grade (Honeywell, Germany; Merck, Darmstadt, Germany). Reference standards for the fifteen quantified compounds (quinic acid, chlorogenic acid, caffeic acid, syringic acid, epicatechin, rutin, o‐coumaric acid, quercitrin, apigenin‐7‐O‐glucoside, rosmarinic acid, salvianolic acid, naringenin, apigenin, luteolin, and acacetin) were of analytical grade and were purchased from Sigma Chemical Co. (St. Louis, MO, USA). Reagents for phytochemical and antioxidant assays, including Folin–Ciocalteu reagent, gallic acid, quercetin, aluminum chloride, sodium carbonate, trichloroacetic acid (TCA), ferric chloride, ferrozine, potassium ferricyanide, ammonium molybdate, neocuproine, copper (II) chloride dihydrate, and phosphate salts—were purchased from Sigma–Aldrich (St. Louis, MO, USA). Reference antioxidants, including butylated hydroxytoluene (BHT), butylated hydroxyanisole (BHA), ascorbic acid, EDTA, and α‐tocopherol, were used as reference antioxidants. For radical‐scavenging assays, 1,1‐diphenyl‐2‐picrylhydrazyl (DPPH^•^) and 2,2′‐azinobis‐(3‐ethylbenzothiazoline‐6‐sulfonic acid) diammonium salt (ABTS^+^^•^) were obtained from Sigma–Aldrich and Fluka (Sternheim, Germany), respectively. Enzyme inhibition assays employed acetylcholinesterase (AChE, Type VI‐S, Electrophorus electricus, EC 3.1.1.7, 425.84 U/mg), butyrylcholinesterase (BChE, equine serum, EC 3.1.1.8, 11.4 U/mg), acetylthiocholine iodide, and butyrylthiocholine chloride, all purchased from Sigma–Aldrich. Galantamine hydrobromide and acarbose were used as positive controls.

### 2.2 Plant Material

Aerial parts of *S. balansae* were collected during the flowering stage from the Aurès Mountains, northeastern Algeria. The plant was taxonomically identified and authenticated by Dr. Zeraib A., a lecturer at Abbes Laghrour University, Khenchela, Algeria. A voucher specimen representing this material has been deposited in the Herbarium of the Biotechnology, Water, Environment and Health Laboratory at Abbes Laghrour University under the code ZA‐SB‐003‐6‐2019. The collected material was washed, shade‐dried at room temperature, and ground into fine powder using a mechanical grinder. The powdered material was stored in airtight containers at ambient conditions until extraction.

### 2.3 Extraction and Yield Calculation

Fifty grams of the powdered aerial parts of *S. balansae* were extracted by maceration in 500 mL of 80% methanol at room temperature with continuous mechanical stirring for 24 h. The same batch of plant material was subjected to three consecutive maceration cycles using fresh solvent for each cycle to ensure exhaustive recovery of phytoconstituents. The filtrates obtained from the three extraction cycles were pooled and concentrated under reduced pressure at 40 °C using a Büchi Rotavapor R‐210 rotary evaporator (Büchi Labortechnik AG, Flawil, Switzerland). The resulting crude extract was transferred to amber vials and stored at 4 °C until further analysis.

The overall extraction yield of the combined crude extract was quantified employing the following formula

Percentage Yield (%) = (Weight of dried crude extract (g) / Weight of initial dried plant material (g)) × 100

This equation allows for the calculation of extraction efficiency by determining the mass proportion of the recovered dried crude extract relative to the initial dry plant material used for extraction.

### 2.4 Phytochemical Screening

Preliminary phytochemical screening of the MESB(methanolic extract of *Salvia balansae*) aerial parts was performed using standard colorimetric and precipitation‐based assays [16, 17]. These tests provided qualitative evidence for the presence of major classes of secondary metabolites, including flavonoids, alkaloids, tannins, saponins, terpenoids, sterols, steroids, phytosteroids, quinones, anthraquinones, coumarins, cardiac glycosides, phlobatannins, anthocyanins, betacyanins, diterpenes, carbohydrates, and reducing sugars. The intensity of characteristic color reactions or precipitate formation was used as an indicator of the presence or absence of each compound group.

### 2.5 Quantitative Analysis

#### 2.5.1 Total Phenolic Content

The total phenolic concentration of the MESB was determined using a spectrophotometric adaptation of the Folin–Ciocalteu assay [18]. Briefly, 200 µL of extract solution (1 mg/mL) was mixed with 1 mL of Folin–Ciocalteu reagent (10% v/v in distilled water). After 4 min, 800 µL of sodium carbonate solution (75 g/L) was added. The reaction was allowed to proceed for 2 h at room temperature in the dark, after which the absorbance was measured at 765 nm using a UV–Vis spectrophotometer (Spectrophotomètre UV–visible 7305 – Jenway). Gallic acid standards (10–200 µg/mL) were used to construct the calibration curve, yielding the regression equation *y* = 0.0099*x* − 0.0773 (*R* ^2^ = 0.9966). Quantification of samples was performed within this validated linear range following blank correction. Results were expressed as µg gallic acid equivalents (GAE) per mg of extract.

#### 2.5.2 Total Flavonoid Content

Flavonoid content was quantified following the aluminum chloride colorimetric procedure [19]. To 0.5 mL of extract solution, 0.5 mL of 2% aluminum chloride in ethanol was added, and the reaction mixture was incubated at room temperature for 1 h. The yellow chromophore formed was measured spectrophotometrically at 420 nm. Quercetin solutions (0–100 µg/mL) served for calibration and provided the calibration equation *y* = 0.013*x* + 0.0426 (*R* ^2^ = 0.9947). Results were reported as µg quercetin equivalents (QE) per mg of extract.

#### 2.5.3 Total Flavonol Content

Flavonols were estimated using the AlCl_3_–sodium acetate assay [20]. Each assay tube contained 0.5 mL extract, 0.5 mL of 2% aluminum chloride solution, and 1.5 mL of 5% sodium acetate. The volume was adjusted to 2.0 mL with ethanol, and the mixture was incubated for 2.5 h at ambient temperature in the dark. Absorbance was recorded at 440 nm, and concentrations were interpolated from a quercetin calibration curve (*y* = 0.0199*x* – 0.0428 and *R* ^2^ = 0.9984). Results were expressed as µg QE per mg of extract.

#### 2.5.4 Condensed Tannins

The condensed tannin content was assessed using the vanillin–hydrochloric acid method [21]. In this assay, 0.5 mL of extract was mixed with 3 mL of 4% vanillin in methanol and 1.5 mL of concentrated hydrochloric acid. After 15 min of reaction at room temperature, absorbance was measured at 500 nm against a reagent blank. Catechin was used for standardization and yielded the regression equation *y* = 0.0022*x* + 0.0066, *R* ^2^ = 0.9914. Results were expressed as µg catechin equivalents (CE) per mg of extract.

#### 2.5.5 Triterpenoid Content

Triterpenoid levels were determined using the vanillin–perchloric acid colorimetric assay [10]. In brief, 0.1 mL of extract was combined with 0.15 mL of vanillin solution in glacial acetic acid and 0.5 mL of perchloric acid. Reaction tubes were incubated at 60 °C for 45 min, cooled rapidly in an ice bath, and then diluted with 2.25 mL of glacial acetic acid. Absorbance was read at 548 nm against a blank. Ursolic acid was employed as a reference (*y* = 0.0004*x* – 0.0138, *R* ^2^ = 0.9975), and concentrations were expressed as µg ursolic acid equivalents (UAE) per mg of extract.

### 2.6 Liquid Chromatography–Mass Spectrometry (LC–MS) Analysis

The phytochemical profile of MESB was determined using a Shimadzu UFLC XR system (Kyoto, Japan), consisting of a CTO‐20AC column oven and an LC‐20ADXR binary pump, coupled to a Shimadzu LCMS‐2020 single quadrupole mass spectrometer (Shimadzu, Kyoto, Japan). Separation was carried out on an Inertsil ODS‐4 C18 column (150 × 3.0 mm, 3 µm) at 40 °C, with a 20 µL injection volume and a flow rate of 0.5 mL/min. Extracts were prepared at a concentration of 1 mg/mL in methanol. Mobile phase A consisted of 0.2% acetic acid in water: methanol (95:5, v/v), and phase B of 0.2% acetic acid in water: acetonitrile (50:50, v/v). The gradient was programmed as: 0–14 min, 10%–20% B; 14–27 min, 20%–55% B; 27–37 min, 55%–100% B; 37–45 min, 100% B; and 45–50 min, re‐equilibration at 10% B.

Mass spectrometry was performed in negative electrospray ionization (ESI) mode under the following conditions: capillary voltage, −3.52 kV; nebulizing gas, 15 L/min; desolvation line, 280 °C; heat block, 400 °C; and detector voltage, 1.35 V. Full‐scan spectra of [M–H]^−^ ions were acquired in the m/z range 100–1000 using the LabSolutions software (Shimadzu, Japan).

Quantification was based on external calibration curves for authentic standards, which showed excellent linearity (*R* ^2^ = 0.99–1.00). Compound identification was confirmed by direct injection of each individual reference standard, using flow injection analysis (FIA) in negative‐ion selected ion monitoring (SIM) mode to confirm the deprotonated molecular ion ([M–H]^−^), followed by chromatographic injection of each standard to determine its retention time. Sample compounds were subsequently identified by matching retention time and m/z with those of the corresponding reference standards. Limits of detection (LOD) and limits of quantification (LOQ) for each analyte, provided by the analytical laboratory, are reported in Table S1. Intraday precision data were not available and therefore cannot be reported [22].

### 2.7 Antioxidant Capacity

The antioxidant potential of MESB was evaluated through a panel of complementary in vitro assays, using a 96‐well format microplate reader (Perkin Elmer EnSpire, Singapore). This assay panel was designed to comprehensively assess the extract's antioxidant potential through complementary mechanisms, encompassing radical scavenging, reducing capacity, and metal chelation.

#### 2.7.1 DPPH Radical‐Scavenging Activity

The free radical‐scavenging activity of MESB was determined using the 2,2‐diphenyl‐1‐picrylhydrazyl (DPPH) assay as described by [23]. In brief, 160 μL of 0.1 mM DPPH solution was mixed with 40 μL of extract at concentrations ranging from 6.25 to 400 μg/mL in a 96‐well plate. After 30 min of incubation in the dark at room temperature, absorbance was measured at 517 nm. The percentage of free radical‐scavenging capacity was calculated according to the following equation

Percentage of free radical activity=(Ac−As)/Ac ×100

where Ac represents the absorbance of the control reaction, and As is the absorbance of the sample reaction mixture.

BHA, BHT, α‐tocopherol, and ascorbic acid served as positive controls. Radical‐scavenging capacity was expressed as IC_50_, defined as the concentration of extract required to quench 50% of DPPH radicals.

#### 2.7.2 ABTS Radical‐Scavenging Activity

The ABTS radical‐scavenging capacity of MESB was evaluated by employing the approach outlined by Re et al. [24]. Radical cations of 2,2′‐azino‐bis(3‐ethylbenzothiazoline‐6‐sulfonate) (ABTS^•+^) were generated by reacting 7 mM ABTS with 2.45 mM potassium persulfate and incubating the mixture for 12–16 h. The solution was diluted to an absorbance of 0.700 ± 0.02 at 734 nm. A mixture of 160 μL of diluted ABTS^•+^ and 40 μL extract (12.5–800 μg/mL) was incubated for 10 min at room temperature, and absorbance was read at 734 nm. The percentage inhibition of the ABTS radical cation by the crude extracts was calculated using the following equation

Percentage of inhibition=[(Ac−As)/Ac] × 100

where Ac indicates the absorbance of the control reaction, and As is the absorbance of the sample reaction mixture.

Standard antioxidants (BHA, BHT, α‐tocopherol, and ascorbic acid) were included. Results were expressed as IC_50_ values.

#### 2.7.3 Cupric Reducing Antioxidant Capacity (CUPRAC)

The reducing ability of the extract was evaluated by the CUPRAC method [25]. Each reaction contained 50 μL neocuproine (7.5 mM), 50 μL CuCl_2_ (10 mM), 60 μL ammonium acetate buffer (pH 7), and 40 μL extract (12.5–800 μg/mL). After 1 h incubation at room temperature in the dark, absorbance was measured at 450 nm. Results were expressed as A_0.5_, the concentration required to reach an absorbance of 0.5.

#### 2.7.4 Superoxide Radical‐Scavenging Activity by Alkaline DMSO Assay

The alkaline DMSO test was adopted to assess the superoxide radical‐scavenging capacity of the tested extract, as outlined by Kunchandy and Rao [26] with slight modifications. Briefly, the reaction mixture consisted of 130 μL alkaline DMSO (prepared by dissolving 20 mg NaOH in 100 mL DMSO), 30 μL NBT solution (1 mg/mL), and 40 μL of extract at concentrations ranging from 12.5 to 800 μg/mL. After mixing, absorbance was measured at 560 nm. The percentage of the scavenging capacity of *S. balansae* was assessed according to the following equation

Superoxide scavenging activity (%)=(Ac−As)/Ac × 100

where Ac represents the absorbance of the control reaction, and As denotes the absorbance of the sample extract.

The results were represented as IC_50_ (µg/mL). BHT and ascorbic acid served as reference antioxidants.

#### 2.7.5 Ferrous Ion‐Chelating Activity

The ability of the extract to chelate ferrous ions was assessed using the ferrozine assay [27]. Briefly, 40 μL of extract solution (12.5–800 μg/mL) was combined with 40 μL methanol, 40 μL FeCl_2_ (0.2 mM), and 80 μL ferrozine reagent. After incubation for 10 min at room temperature, absorbance was measured at 593 nm. The percentage of ferrous ions (Fe^2+^) chelated by the extracts was quantified using the following formula

$${\mathrm{Fe}}^{2+}\;\mathrm{chelating}\;(\%)\;=\;(\mathrm{Ac}\;-\;\mathrm{As})/\mathrm{Ac}\;\times \;100$$

where Ac indicates the absorbance of the control reaction, and As is the absorbance of the sample extract.

The results were represented as IC_50_ (µg/mL). EDTA and α‐tocopherol served as reference chelators.

#### 2.7.6 Ferric Reducing Antioxidant Power (FRAP)

The reducing power of the extracts was determined according to the potassium ferricyanide reduction method [28]. Different concentrations of the extract (3.125–200 μg/mL) were mixed with 40 μL phosphate buffer (0.2 M, pH 6.6) and 50 μL potassium ferricyanide (1%). Following incubation at 50 °C for 20 min, 50 μL trichloroacetic acid (10%) was added to terminate the reaction. The mixture was then diluted with 40 μL distilled water and 10 μL FeCl_3_ (0.1%). Absorbance was recorded at 700 nm, and reducing power was expressed as A_0.5_, the concentration required to achieve an absorbance of 0.5. BHA, BHT, ascorbic acid, and α‐tocopherol were used as standard references.

#### 2.7.7 Phenanthroline Assay

Chelating activity was further quantified using the 1,10‐phenanthroline assay, which measures the ability of extracts to reduce Fe^3+^ to Fe^2+^ [29]. Reaction mixtures consisted of 10 μL extract (3.125–200 μg/mL), 50 μL FeCl_3_ (0.2%), 30 μL 1,10‐phenanthroline (0.5%), and 110 μL methanol. After 10 min of incubation at 20 °C, absorbance was measured at 510 nm. Results were expressed as A_0.5_ values. Reference standards used were BHA, ascorbic acid, and BHT.

#### 2.7.8 Silver Nanoparticle Antioxidant Capacity (SNPAC)

The capacity of the extract to reduce silver ions and stabilize nanoparticles was assessed using the SNP assay [30]. Extract solutions (3.125–400 μg/mL) were mixed with 50 μL distilled water and 130 μL of freshly prepared SNP reagent (1 mM AgNO_3_ in 1% sodium citrate) in 96‐well plates. After incubation at 25 °C for 30 min, absorbance was measured at 423 nm, corresponding to the surface plasmon resonance of silver nanoparticles. Antioxidant capacity was expressed as A_0.5_ values, with Trolox and ascorbic acid used as positive controls.

### 2.8 In Vitro Anti‐Inflammatory Activity

The anti‐inflammatory potential of MESB was evaluated using the bovine serum albumin (BSA) protein denaturation model [31]. A 0.5 mL aliquot of 0.2% BSA solution prepared in Tris‐buffered saline (pH 6.8) was mixed with 0.5 mL of extract solution at varying concentrations. The mixture was incubated at 37 °C for 15 min and then subjected to thermal denaturation at 72 °C for 5 min. Following cooling to room temperature (10 min), absorbance was measured at 660 nm using a microplate reader. The ability of the extract to inhibit protein denaturation was expressed as a percentage of inhibition, calculated using the following equation

% Inhibition = ((Absorbance of Control − Absorbance of Sample)/Absorbance of Control) × 100

Diclofenac sodium was used as the reference standard for comparison.

### 2.9 α‐Amylase Inhibition Assay

The in vitro antidiabetic activity of MESB was determined using the α‐amylase inhibition assay, following the procedure of Zengin & Aktumsek [32] with slight modifications. Briefly, 25 µL of the extract or the standard inhibitor (acarbose) at different concentrations was mixed with 50 µL of α‐amylase solution (1 U/mL in sodium phosphate buffer, pH 6.9, containing 6 mM NaCl) and preincubated at 37 °C for 10 min. The reaction was initiated by adding 50 µL of 1% soluble starch solution, followed by incubation at 37 °C for 20 min. The reaction was terminated by the addition of 25 µL of 1 M HCl, after which 100 µL of iodine–potassium iodide solution was added to facilitate color development. A blank control was prepared under identical conditions without the enzyme. Absorbance was measured at 630 nm, and the percentage inhibition of α‐amylase activity was calculated according to the following equation

α‐amylase inhibition(%)=1 − [(Ac − Ae) − (As − Ab)/(Ac − Ae)]

where Ac indicates the absorbance of control (starch + iodine reagent + HCl + buffer, without enzyme or extract), Ae the absorbance of enzyme control (starch + α‐amylase + iodine reagent + HCl, without extract), As the absorbance of sample (starch + α‐amylase + extract + iodine reagent + HCl), and Ab the absorbance of sample blank (extract + iodine reagent + buffer, withoutenzyme).

### 2.10 Anticholinesterase Activity

The inhibitory activities of *S. balansae* extract against acetylcholinesterase (AChE) and butyrylcholinesterase (BChE) were assessed spectrophotometrically using the Ellman method with a 96‐well microplate reader [33]**.** Briefly, 10 µL of MESB, or galantamine as a reference standard, prepared in ethanol at different concentrations, was mixed in microplate wells with 150 µL of sodium phosphate buffer (100 mM, pH 8.0) and 20 µL of enzyme solution (AChE, 5.32 × 10^−3^ U; BChE, 6.85 × 10^−3^ U). After 15 min of incubation at 37 °C, 10 µL of 5,5′‐dithiobis‐(2‐nitrobenzoic acid) (DTNB, 0.5 mM) was added, followed by 10 µL of acetylthiocholine iodide (0.71 mM) for AChE or 10 µL of butyrylthiocholine chloride (0.2 mM) for BChE as the respective substrates. Absorbance was measured at 412 nm at baseline (0 min) and after 15 min of incubation at 37 °C. The percentage inhibition was calculated relative to the control, and IC_50_ values were derived for the extracts and galantamine.

### 2.11 Acute Oral Toxicity Test

The acute oral toxicity of the methanolic plant extract was assessed using a limit test based on OECD Guideline 423 [34], with all procedures approved by the institutional animal ethics committee (Reference No. 03/2024). Wistar rats (male, 6–8 weeks, 150–200 g) were obtained from the Institut Pasteur d’Algérie (Algiers, Algeria) and acclimatized under standard laboratory conditions with free access to food and water.

Animals were randomly divided into two groups (*n* = 6 per group). The treated group received a single oral dose of 2000 mg/kg of the extract suspended in distilled water, while the control group received vehicle alone. Following administration, animals were closely monitored for the first 4 h, then at 24 h, and once daily for 14 days. Clinical signs including mortality, changes in physical appearance, salivation, lethargy, or other behavioral alterations were systematically recorded. On day 15, all animals were sacrificed. The liver and kidneys were excised, weighed, and relative organ weights were calculated as

Relative organ weight (%) = (organ weight (g) / body weight on sacrifice day (g)) × 100

For histopathology, tissues were fixed in 10% neutral‐buffered formalin, processed by standard paraffin embedding, sectioned at 5 µm, and stained with hematoxylin and eosin (H&E). Sections were examined under a light microscope (Optika DM‐25, Italy) by a pathologist blinded to the treatment groups, and representative micrographs were captured for documentation.

### 2.12 Croton Oil‐Induced Ear Edema Assay

**FIGURE 1:**
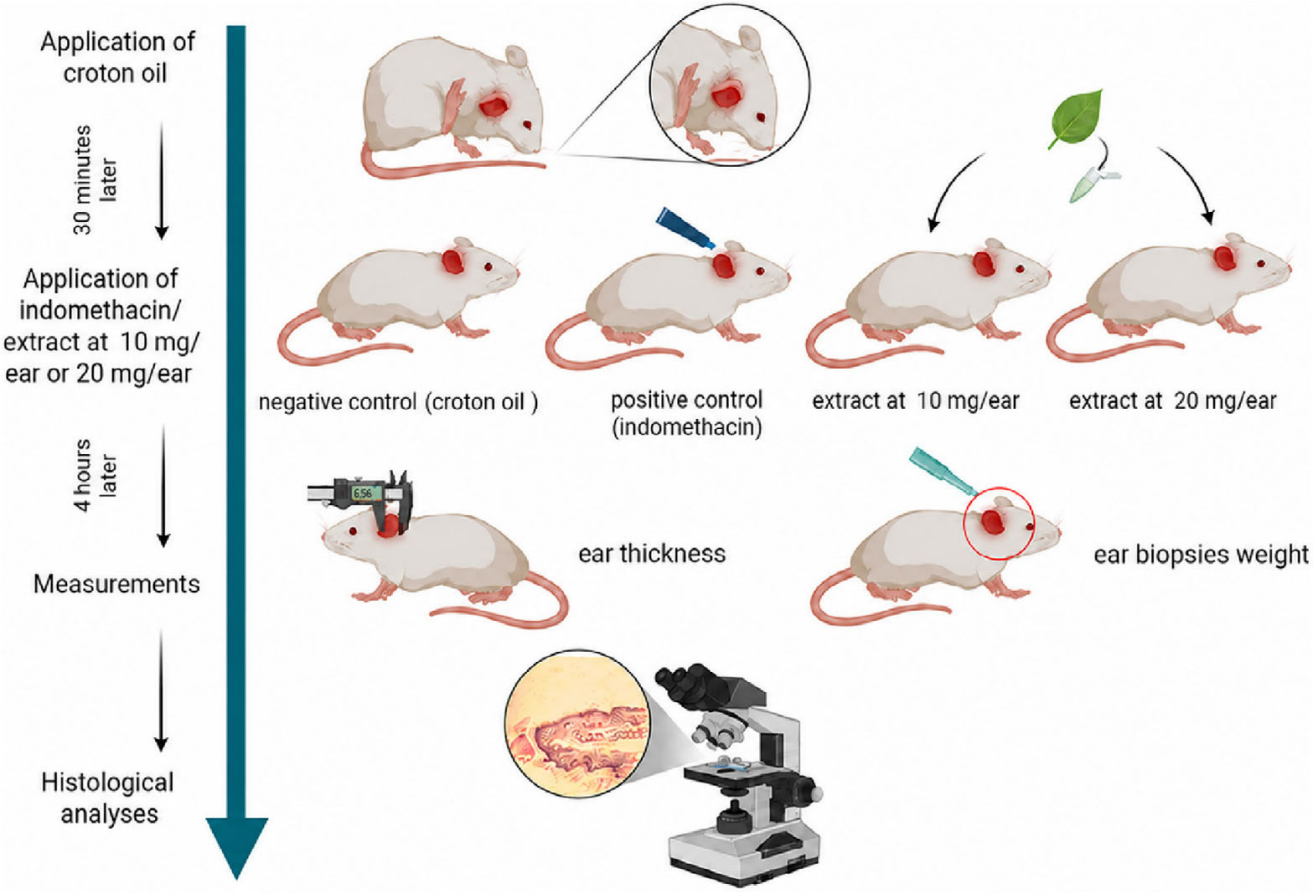
Experimental design of the croton oil‐induced ear edema model.

The croton oil‐induced ear edema model was employed to evaluate the topical anti‐inflammatory activity of the extract, following the method of Londonkar et al. [35] with slight modifications. A solution of 40 μL croton oil (35% in acetone) was applied topically to the right ear of Wistar rats, while the left ear was left untreated as a control. The plant extract (10 and 20 mg/ear in acetone) and indomethacin (reference drug) were applied 30 min after croton oil administration. Ear thickness was measured 4 h post‐induction using a digital caliper. At the end of the experiment, 6 mm ear biopsies were excised and weighed (Figure 1). Percentage inhibition of edema was calculated using the equation

Inhibition % = [(Wc ‐ Wt)/Wc] × 100

where Wc represents the mean edema weight in the negative control group, and Wt denotes the mean edema weight in each treated group.

Ear biopsies were fixed in 10% neutral buffered formalin for 24 h, then dehydrated, embedded in paraffin, and sectioned at 5 µm using a microtome. The cross‐sections were stained with hematoxylin and eosin (H&E) for the evaluation of leukocyte infiltration and edema intensity. For quantitative histomorphometric analysis, slides were examined under a light microscope (Optika DM‐25, Italy). Total ear thickness and epidermal thickness were measured manually on the stained cross‐sections at ×100 magnification using the ImageJ software (version 1.53t, NIH, USA). To eliminate observer bias, all image captures and subsequent morphometric measurements were performed by a pathologist blinded to the treatment groups. Five randomly selected fields per section were analyzed per animal, and mean thickness values were calculated for each experimental group.

### 2.13 Molecular Docking

The crystal structure of the protein NLRP3 was obtained from the PDB Database (https://www.rcsb.org/) with ID 7ALV [36]. After removing cocrystalized ligands and water molecules via BIOVIA Discovery Studio Visualizer [37], the protein was preprocessed by the AutoDock Tools 1.5.7 software: hydrogen atoms and Kollmancharges were added.

The structures of the phytocompounds identified in *S. balansae* extract, as well as reference molecules MCC950 (reference inhibitor) and RM5 (native ligand), were downloaded from the PubChem data bank (https://pubchem.ncbi.nlm.nih.gov/) and then submitted to optimization and conformational search (MMFF94) using the AVOGADRO software [38].

Molecular docking was performed against the active site of NLRP3 (PDB: 7ALV) using AutoDock Vina as implemented in PyRx [39]. The docking grid box was centered at coordinates *x* = 16.69, *y* = 34.24, *z* = 125.27 Å, with dimensions of 21.79 × 23.80 × 21.42 Å, encompassing all key binding site residues. The exhaustiveness parameter was set to 8. Validation of the docking protocol was performed by redocking the cocrystalized ligand RM5 into the binding site; the RMSD between the redocked and crystallographic poses was 1.8 Å (<2.0 Å), confirming the reliability of the protocol. Docking poses and molecular interactions were visualized using BIOVIA Discovery Studio Visualizer (Dassault Systèmes BIOVIA, 2023). Protein–ligand interaction distances were analyzed using the interaction analysis tools implemented in BIOVIA Discovery Studio Visualizer and are expressed in angstroms (Å).

### 2.14 Statistical Analysis

All results were expressed as mean ± standard deviation (SD). Statistical analyses were performed using an unpaired Student's *t*‐test. A *p*‐value < 0.05 was considered statistically significant. Statistical significance among multiple groups was determined by one‐way analysis of variance (ANOVA) followed by the least significant difference (LSD) post hoc test to compare mean values. GraphPad Prism (version 8.0.2, GraphPad Software, San Diego, CA, USA) was used for all calculations and graphical representations.

## 3 Results and Discussion

### 3.1 Extraction Yield

The extraction of phenolic compounds from the endemic Algerian species *S. balansae* was investigated using simple maceration with methanol, yielding 22.48% from the aerial parts (Table 1). This yield indicates an efficient recovery rate of bioactive compounds from the plant material.

**TABLE 1:** Phytochemical composition of *Salvia balansae* methanolic aerialparts extract.

Phytochemical component	Content (mean ± SD)
Extraction yield, %	22.48
Total protein µg/mg extract	12.125 ± 0.375
Total phenolic content, µg GAE/mg	116.014 ± 0.514
Total flavonoid content, µg QE/mg	70.262 ± 0.347
Total flavonols content, µg QE/mg	28.147 ± 0.874
Condensed tannin content, µg CE/mg	6.370 ± 0.642
Triterpenoid contents, µg UAE/mg	124.778 ± 1.443

*Note:* All values are presented as the mean ± standard deviation (*n* = 3).

When compared with previous findings by Bendrihem et al. [14], who reported differential extraction yields from various plant parts of *S. balansae*—flowers (29.99%), leaves (20.05%), and stems (12.34%)—our yield of 22.48% from the aerial parts represents an intermediate value, which is logical given that our extract encompasses a mixture of these plant parts. The variation in extraction yields between plant parts suggests a nonuniform distribution of extractable compounds throughout the plant, with flowers being particularly rich in methanol‐soluble compounds.

Comparatively, *S. balansae* shows similar yields to *S. officinalis* (20.7%–25.2%) and *S. verbenaca* (12.1%–24.4%) but exceeds the yields reported for *S. aegyptiaca* (12.6%–14.5%) and *S. argentea* (10.3%–12.3%) [40]. Such differences underscore species‐specific variations in metabolite composition influenced by genetics, environmental factors, and harvesting conditions. The extraction yield, particularly through maceration, depends on various factors, including solvent type, temperature, and extraction time [41].

### 3.2 Phytochemical Screening

The qualitative phytochemical screening of MESB, as shown in Table 2, revealed the presence of a diverse array of secondary metabolites, providing insights into its chemical composition and potential biological activities. The results indicated the presence of several important classes of compounds, including flavonoids, tannins, saponins, terpenoids, quinones, cardiac glycosides, sterols, phytosteroids, coumarins, betacyanins, and diterpenes, while confirming the absence of alkaloids, anthraquinones, steroids, anthocyanins, and phlobotannins.

**TABLE 2:** Qualitative phytochemical profile of *S. balansae* methanolic aerial parts extract.

Compounds	Results
1‐Flavonoids	
‐Shinoda test	+
‐FeCl_3_ test	+
‐Alkaline reagent test	+
2‐Alkaloids	
‐Dragendorff's test	−
‐Mayer's test	−
3‐Tannins	
‐FeCl_3_ test	+
‐Alkaline reagent test	+
4‐Saponins	+
5‐Terpenoids	+
6‐Quinones	+
7‐Anthraquinones	−
8‐Cardiac glycosides	+
9‐Sterols	+
10‐Steroids	−
11‐Phytosteroids	+
12‐Coumarins	+
13‐Anthocyanins	−
14‐Betacyanins	+
15‐Phlobotannins	−
16‐Diterpenes	+
17‐Carbohydrates	+
18‐Reducing sugars	+

*Note*: (+) Presence; (−) Absence.

These findings align with prior research on the *Salvia* genus, which consistently highlights its richness in bioactive compounds, particularly flavonoids and terpenoids, both recognized for their therapeutic effects [42, 43].

Similarly, Mokhtar et al. [4] identified saponins, reducing sugars, and quinones in aqueous extracts of *S. balansae*, further emphasizing the phytochemical diversity and richness of this endemic species. Additionally, the phytochemical screening of the aqueous leaf extract of *S. balansae* by Mekki et al. [12] reported the presence of polyphenols, flavons, catechols, tannins (catechic and gallic acids), amino acids, proteins, cardiac glycosides, and saponins, with the absence of flavonons, leucoanthocyanins, anthocyanins, phlobatannins, oxalate, terpenoids, and alkaloids. These findings complement the present study, which also identified flavonoids, tannins, saponins, and cardiac glycosides in the methanolic extract. The consistent identification of similar compounds across studies supports the phytochemical richness of *S. balansae* while highlighting differences that may arise from variations in extraction methods or environmental conditions.

Flavonoids and tannins are well known for their antioxidant and anti‐inflammatory properties [44], while saponins and terpenoids exhibit immunomodulatory, anticancer, and antimicrobial activities [45, 46]. The presence of cardiac glycosides and sterols indicates potential cardioprotective and anti‐inflammatory benefits. Interestingly, the absence of alkaloids, anthocyanins, and phlobatannins reflects species‐specific biosynthetic pathways, potentially avoiding the toxicity sometimes associated with alkaloids [47].

Compared to other *Salvia* species, *S. balansae* demonstrates comparable phytochemical diversity, reinforcing its potential as a rich source of bioactive compounds. These findings highlight its promise for developing antioxidant, anti‐inflammatory, and antimicrobial agents, supporting further investigation of its therapeutic potential.

### 3.3 Quantitative Analysis

#### 3.3.1 Total Phenolic Content

The total phenolic content (TPC) is a crucial parameter for evaluating the potential bioactivity and therapeutic applications of plant extracts. Phenolic compounds are well‐recognized for their antioxidant properties, which are primarily related to the presence, position, and molecular size of hydroxyl groups in their chemical structures [48].

In the present study, the total phenolic content of MESB was determined to be 116.01 ± 0.51 µg GAE/mg, as shown in Table 1.

The TPC observed in our study is broadly consistent with findings from other research on *S. balansae*, though some differences are apparent. Bendrihem et al. [14] reported values ranging from 99.85 ± 0.10 µg GAE/mg in stems to 147.87 ± 0.21 µg GAE/mg in flowers of *S. balansae*. Similarly, Mekki et al. [12] recorded 134.91 ± 0.23 mg GAE/g in aqueous extracts of *S. balansae*, while Mahdjoub et al. [11] reported an average TPC of 49.63 ± 6.31 mg GAE/g DW for methanolic leaf extracts. Variations across studies may be explained by differences in plant parts, extraction methods, and solvent polarity, as methanol efficiently recovers both polar and semipolar compounds [41].

Compared to other *Salvia* species, the total phenolic content of *S. balansae* is higher than the reported ranges for *Salvia officinalis* (24.52–95.62 mg GAE/g) and *S. sclarea* (29.39–91.02 mg GAE/g) [49], indicating its relatively greater phenolic richness. However, it is slightly lower than the total phenolic content of *S. bracteata* (121.66 mg GAE/g) [50] and significantly lower than the exceptionally high TPC of aqueous extracts of *S. lavandulifolia* (246.51 mg GAE/g) [51], which may be due to differences in extraction solvents, environmental factors, and species‐specific biosynthesis.

These interspecific variations likely reflect phylogenetic and evolutionary adaptations to different ecological niches [52], with the relatively high phenolic content in *S. balansae* suggesting enhanced biosynthesis of phenolic compounds via the phenylpropanoid pathway, likely as an adaptation to environmental stressors in its native habitat [53].

The substantial total phenolic content has important therapeutic implications, as phenolic compounds can act as hydrogen donors, singlet oxygen quenchers, and metal chelators, indicating strong antioxidant potential, particularly relevant for developing natural therapeutic agents against oxidative stress‐related disorders [54].

#### 3.3.2 Total Flavonoid and Flavonol Content

Flavonoids and flavonols are pivotal phytochemicals in *Salvia* species, contributing significantly to their antioxidant and anti‐inflammatory properties [55].

The total flavonoid content (TFC) and total flavonol content (FOL) of the studied plant extract were 70.26 ± 0.35 and 28.15 ± 0.87 µg QE/mg, respectively, as shown in Table 1.

When compared to prior research on *S. balansae*, our findings are in agreement with Bendrihem et al. [14], who reported total flavonoid values ranging from 16.67 ± 0.20 µg QE/mg in stems to 72.17 ± 0.12 µg QE/mg in leaves and flavonol values of up to 35.28 ± 0.05 µg QE/mg in ethanolic leaf extracts. However, Mahdjoub et al. [11] and Mekki et al. [12] recorded lower flavonoid levels, with 5.96 ± 0.004 mg QE/g in aqueous extracts and 8.14 ± 0.39 mg QE/g DW in methanolic leaf extracts, respectively. These differences can be attributed to variations in plant parts, extraction solvents, and geographic origins. The higher flavonoid levels in our samples and those of Bendrihem et al. [14] where from the Aurès region (semiarid continental climate), compared to Mahdjoub et al. [11] and Mekki et al. [12] samples from the Cheliff Valley's milder maritime climate, demonstrate the significant influence of environmental conditions on secondary metabolite production. Semiarid conditions typically induce higher UV exposure and oxidative stress, triggering enhanced flavonoid biosynthesis [56, 57]. This environmental adaptation mechanism involves the upregulation of key enzymes such as phenylalanine ammonia‐lyase (PAL) and chalcone synthase (CHS) under stress conditions [53, 58].

Compared to other *Salvia* species, *S. balansae* exhibits notable flavonoid and flavonol profiles. For instance, *Salvia hierosolymitana* and *S. eigii* exhibit exceptionally high TFCs of 770.85 mg QE/g DW and 520.60 mg QE/g DW, respectively [59], while moderate values are reported for *S. officinalis* (13.73–55.38 mg QE/g) [50]. Flavonol levels similarly vary, with *Salvia pisidica* extract containing 18.19 mg rutin equivalents (RE)/g [60], while *S. sclarea* flower extracts subjected to maceration yielded 9.20 ± 0.84 mg QE/g, compared to lower levels of 5.45 ± 0.01 mg QE/g obtained via rotary evaporator (rotavapor) extraction [61]. These findings emphasize the impact of extraction methods, with maceration being particularly effective in retaining flavonoids and flavonols, as demonstrated in our study.

These findings highlight not only the significant impact of extraction methodology and environmental conditions on flavonoid and flavonol content but also underscore the remarkable adaptability of *Salvia* species through modulation of their secondary metabolite profiles. The high flavonoid content in *S. balansae*, particularly under semiarid conditions, suggests an evolved capacity for enhanced flavonoid biosynthesis as an adaptive response to environmental stress. This characteristic, combined with the significant proportion of flavonols, positions *S. balansae* as a promising source of bioactive compounds with potential applications in pharmaceutical and nutraceutical industries.

#### 3.3.3 Condensed Tannin Content

Condensed tannins (CT), or proanthocyanidins, represent a significant class of polyphenolic compounds characterized by their polymeric flavan‐3‐ol units. These compounds have garnered substantial attention in phytochemical research due to their diverse biological activities [44].

In our investigation, MESB demonstrated a condensed tannin content of 6.37 ± 0.64 µg CE/mg (Table 1), which quantitatively supports the results of the qualitative phytochemical screening and indicates the presence of tannins. In contrast, Mekki et al. [12] reported a considerably higher condensed tannin concentration (19.16 ± 2.5 mg CE/g) in aqueous extracts of *S. balansae* leaves. The discrepancy in tannin levels can be attributed to differences in plant part selection, extraction solvent, and ecological origin.

The selection of plant tissue plays a pivotal role in determining phytochemical composition. Leaves, as investigated by Mekki et al. [12], are well‐documented sites of polyphenol accumulation and often exhibit higher levels of condensed tannins compared to mixed aerial parts [62, 63]. Although hydroalcoholic solvents such as methanol are generally more effective for extracting tannins, the elevated tannin content reported by Mekki may be better explained by environmental influences. Their plant material was collected from the Cheliff Valley, a temperate maritime region where favorable climatic conditions may upregulate the biosynthesis of phenolic compounds. In contrast, our samples originated from the Aurès Mountains, a high‐altitude, semiarid environment characterized by harsher abiotic stressors that could modulate secondary metabolism differently.

Supporting this variability, Khiya et al. [64] reported a wide range of condensed tannin levels (0.42–162.53 mg CE/g) in *S. officinalis* leaf extracts from different Moroccan regions, with hydromethanolic and aqueous fractions showing particularly high values. Their results were positively correlated with antioxidant capacity, emphasizing the functional relevance of CTs across diverse extraction conditions and geographic settings.

Further interspecific comparisons reinforce the impact of methodological and ecological variables. For instance, in Tunisian populations of *S. officinalis*, Jedidi et al. [65] observed CT values ranging from 19.48 ± 2.58 to 28.66 ± 1.17 mg CE/g in aqueous extracts, attributing this variation to environmental factors such as soil pH, altitude, and regional climate. Similarly, *S. lavandulifolia* aqueous leaf extracts examined by Remok et al. [51] exhibited lower CT levels (2.46 ± 0.08 mg CE/g DW), yet still displayed potent antioxidant and antihyperglycemic properties. These findings suggest that even moderate concentrations of condensed tannins can confer meaningful biological activity, particularly in metabolic and oxidative stress‐related pathways.

These comparative analyses affirm that condensed tannin accumulation in *Salvia* species is not only species‐specific but also highly responsive to both extraction methodology and environmental conditions. The presence of CTs in *S. balansae*, even at moderate levels, contributes substantively to its antioxidant profile and supports its continued exploration for therapeutic applications targeting oxidative and metabolic dysfunction.

#### 3.3.4 Total Triterpenoid Content

Triterpenoids represent a crucial class of secondary metabolites, widely recognized for their anti‐inflammatory, hepatoprotective, and anticancer activities [66]. In our study, the methanolic extract of *S. balansae* aerial parts exhibited a notably high total triterpenoid content, quantified at 124.78 ± 1.44 µg UAE/mg of extract (Table 1). This substantial value, measured using a colorimetric method, reflects a notable accumulation of triterpenoid compounds and aligns with the phytochemical richness previously observed in other *Salvia* species [67, 68].

While comprehensive studies on total triterpenoid content (TTC) remain scarce in the *Salvia* genus, Triterpenoid quantification in *Salvia* species has primarily employed chromatographic techniques such as HPLC and LC‐MS to identify and measure key triterpenic acids, notably ursolic acid (UA), oleanolic acid (OA), and betulinic acid (BA). For instance, Abdollahi‐Ghehi et al. [69] reported UA contents as high as 4.34 ± 0.1 mg/g DW in *S. officinalis* and 3.71 ± 0.08 mg/g DW *in S. multicaulis*, while OA and BA levels ranged between 0.06 and 1.96 mg/g DW and 0.17 and 3.12 mg/g DW, respectively. Compared to these targeted values, the total triterpenoid concentration observed in *S. balansae* suggests a broader spectrum of triterpenes beyond the classical UA, OA, and BA, or a higher cumulative presence of these compounds.

The elevated TTC observed in *S. balansae* likely reflects adaptive metabolic responses to its native environment. Collected from the high‐altitude, semiarid Aurès Mountains, this species is exposed to abiotic stresses, such as increased solar radiation, low water availability, and temperature extremes, that are known to upregulate triterpenoid biosynthesis as a defensive strategy [70]. These stressors may explain the notably higher triterpenoid levels compared to cultivated *Salvia* species from more temperate or maritime environments.

Furthermore, the accumulation of triterpenoids in *S. balansae* could serve as a chemotaxonomic marker, supporting its phylogenetic relationship with other medicinal *Salvia* species, especially within the section *Aethiopis*, which is recognized for its high levels of terpenoid derivatives [68, 71]. The distinct TTC observed in *S. balansae* may therefore serve as a phytochemical signature of its species or population, reinforcing its uniqueness within the genus.

Pharmacologically, the triterpenoid richness of *S. balansae* holds considerable promise. Numerous triterpenes from *Salvia* species, particularly UA and BA, are well‐documented for their antioxidant, anti‐inflammatory, hepatoprotective, and anticancer properties [70]. Given the abundance of triterpenoids observed in this study, *S. balansae* may represent a potent candidate for therapeutic development, particularly in the context of oxidative stress‐ and inflammation‐related pathologies.

To fully elucidate the triterpenoid profile of *S. balansae*, future studies should employ advanced chromatographic techniques, such as high‐performance liquid chromatography coupled with mass spectrometry (HPLC‐MS), to identify and quantify individual triterpenoid constituents.

### 3.4 LC‐MS‐Based Phytochemical Profiling of *S. balansae*

The LC‐MS analysis of MESB revealed a complex phytochemical profile composed primarily of polyphenolic compounds, including phenolic acids and flavonoids. As presented in Table 3, a total of 15 major compounds were identified and quantified, showcasing a high abundance of flavones and hydroxycinnamic acid derivatives.

**TABLE 3:** Identified compounds in the methanolic extract of *S. balansae* by LC‐MS analysis.

No.	Compound name	Retention time, min	m/z	Molecular formula	Concentration, µg/g dry extract	Phytochemical class	ID level^a^
1	Quinic acid	1.436	191.00	C_7_H_11_O_6_	59.73	Phenolic acid	1
2	Chlorogenic acid	1.588	353.00	C_16_H_18_O_9_	0.41	Phenolic acid	1
3	Caffeic acid	2.144	179.00	C_9_H_8_O_4_	40.66	Phenolic acid	1
4	Syringic acid	2.148	197.00	C_9_H_10_O_5_	58.47	Phenolic acid	1
5	Epicatechin	1.544	289.00	C_15_H_14_O_6_	16.66	Flavanol	1
6	Rutin	1.468	609.00	C_27_H_30_O_16_	8.49	Flavonol glycoside	1
7	o‐Coumaric acid	2.304	163.00	C_9_H_8_O_3_	2.20	Phenolic acid	1
8	Quercitrin (Quercetin‐3‐o‐rhamnoside)	2.101	447.00	C_21_H_20_O_11_	88.67	Flavonol glycoside	1
9	Apigenin‐7‐O‐glucoside	2.071	431.00	C_21_H_20_O_10_	50.87	Flavone glycoside	1
10	Rosmarinic acid	1.480	359.00	C_18_H_16_O_8_	107.76	Polyphenolic ester	1
11	Salvianolic acid B	1.461	717.00	C_36_H_30_O_16_	6.78	Caffeic acid	1
12	Naringenin	3.295	271.00	C_15_H_12_O_5_	6.95	Flavanone	1
13	Apigenin	3.294	269.00	C_15_H_10_O_5_	22.48	Flavone	1
14	Luteolin	2.634	285.00	C_15_H_10_O_5_	720.70	Flavone	1
15	Acacetin	6.718	283.00	C_16_H_12_O_5_	8.64	Flavone	1

*Note:* Concentrations expressed as µg/g dry extract.

a Identification levels follow the Metabolomics Standards Initiative (MSI) criteria [72]. Level 1 (identified metabolite): confirmed by comparison of retention time and accurate mass ([M–H]^−^) with an authentic reference standard analyzed following the same analytical procedure applied to all analytes in this study.

Among the identified constituents, luteolin was the most abundant metabolite (720.70 ppm), followed by rosmarinic acid (107.76 ppm), quercitrin (quercetin‐3‐O‐rhamnoside) (88.67 ppm), and apigenin‐7‐O‐glucoside (50.87 ppm). These molecules are widely recognized for their potent antioxidant, anti‐inflammatory, and cytoprotective properties [73]. The exceptionally high concentration of luteolin is particularly notable, as it contrasts with the typical phenolic profile of *Salvia* species, where rosmarinic acid is usually the quantitatively dominant compound, especially in polar‐solvent extracts [8, 74]. Nevertheless, flavones such as luteolin and apigenin, particularly in their glycosylated forms like 7‐O‐glucosides, are widely distributed across the genus and serve as reliable chemotaxonomic markers [2, 75]. The unusual predominance of luteolin in *S. balansae* suggests a distinctive chemotype, potentially reflecting ecological or evolutionary adaptations specific to its endemic range in the Aurès Mountains.

Phenolic acids constituted a significant portion of the extract, with quinic acid (59.73 ppm), syringic acid (58.47 ppm), and caffeic acid (40.66 ppm) detected in substantial concentrations. Rosmarinic acid, a caffeic acid ester derivative characteristic of the Lamiaceae family, particularly the *Salvia* genus, exhibited the second‐highest concentration (107.76 ppm) among all identified compounds. These compounds have been extensively studied for their potent antioxidant properties and are considered a chemotaxonomic marker for this genus within the Lamiaceae family [76].

Flavonoids emerged as the most abundant and structurally diverse group among the identified metabolites in the *S. balansae* extract, encompassingflavones, flavonols, and flavanones. Such diversity is consistent with the metabolic patterns reported in *Salvia* and underscores the evolutionary optimization of polyphenol biosynthesis in this genus. For instance, a broad screening of 50 Iranian *Salvia* species revealed recurrent detection of compounds such as rutin, luteolin‐7‐O‐glucoside, and salvianolic acid B [77]. In contrast, studies of Mediterranean and Eastern European species, including *S. sclarea*, *S. verticillata*, *S. absconditiflora*, and *S. fruticose*, highlight substantial variation in both phenolic composition and metabolite dominance [78, 79]. These molecules contribute to oxidative homeostasis through radical scavenging and metal chelation and via modulation of signaling pathways, including NF‐κB and MAPK. Glycosylated derivatives such as quercitrin and apigenin‐7‐O‐glucoside are particularly relevant due to their enhanced solubility and potential bioavailability.

Salvianolic acid B, a caffeic acid oligomer detected at 6.78 ppm, represents another characteristic compound of the genus *Salvia*. This water‐soluble polyphenolic compound, composed of three caffeic acid units, has been extensively studied in *Salvia miltiorrhiza*, where it demonstrates potent antioxidant, anticancer, and neuroprotective effects through multiple mechanisms, further reinforcing the pharmacological value of the extract [80, 81]. Its presence in *S. balansae*, albeit at a relatively modest concentration, suggests the conservation of biosynthetic pathwaysacross *Salvia* species.

The comparative phytochemical analysis supports the chemotypic distinctiveness of *S. balansae* from the Aurès Mountains. The findings are consistent with Bendrihem et al. [14], whose study also focused on *S. balansae* from the Aurès region. Their LC‐MS data revealed high levels of luteolin‐7‐O‐glucoside, apigenin‐7‐O‐glucoside, and rosmarinic acid in various plant parts, supporting the idea of an Aurès‐specific chemotype rich in flavone derivatives and hydroxycinnamic acids. This contrasts markedly with studies conducted on *S. balansae* populations from theMostaganem region [4, 11], where catechin, myricetin, tannic acid, benzoic acid, and gallic acid were reported as the dominant compounds. Such discrepancies may reflect ecotypic divergence driven by genetic variation or regional environmental pressures and highlight the need for integrative approaches combining metabolomics, genomics, and biogeography. This discrepancy likely reflects underlying microclimatic, edaphic, or genetic variability, a phenomenon well‐documented in *Salvia* species [2, 78].

This comprehensive LC‐MS analysis has provided valuable insights into the phytochemical composition of MESB, revealing a diverse array of bioactive constituents with potential applications in pharmacological research.

### 3.5 Antioxidant Potential of *Salvia Balansae*

Oxidative stress, a pathological hallmark of numerous chronic diseases, results from an imbalance between reactive oxygen species (ROS) production and antioxidant defenses. Therefore, the characterization of natural antioxidants capable of interrupting radical chain reactions, chelating redox‐active metals, and restoring redox equilibrium is of significant biomedical interest. In this context, MESB was subjected to a panel of complementary in vitro antioxidant assays (Table 4), each probing distinct mechanistic aspects of antioxidant activity.

**TABLE 4:** In vitro antioxidant activities of *Salvia balansae* methanolic extract.

	DPPH radical scavenging (IC_50_, µg/mL)	ABTS radical scavenging (IC_50_, µg/mL)	Fe^2+^ chelating assay (IC_50_, µg/mL)	CUPRAC capacity (A_0.5_, µg/mL)	O‐phenantroline assay (A_0.5_, µg/mL)	SNPAC assay (A_0.5_, µg/mL)	O_2_^−^• scavenging alkaline DMSO (IC_50_ µg/ml)
*S. balansae* extract	34.20 ± 0.67^a^	33.12 ± 0.52^a^	78.19 ± 1.79^a^	52.18 ± 0.62^a^	93.36 ± 0.52^a^	118.81 ± 0.99^a^	690.75 ± 0.43^a^
BHA	4.79 ± 0.05^d^	1.86 ± 0.03^d^	NT	5.21 ± 0.37^e^	1.09 ± 0.29^d^	NT	NT
BHT	17.41 ± 0.41^b^	2.59 ± 0.14^c^	NT	9.11 ± 0.16^d^	11.49 ± 0.97^b^	NT	23.73 ± 1.11^b^
α‐Tocopherol	12.95 ± 0.19^c^	8.31 ± 0.49^b^	31.51 ± 0.78^b^	20.25 ± 0.37^b^	NT	NT	NT
Ascorbic acid	13.14 ± 0.12^c^	3.25 ± 0.51^c^	NT	10.66 ± 0.45^c^	9.04 ± 0.90^c^	8.74 ± 0.67^b^	NT
EDTA	NA	NA	8.14 ± 0.64^c^	NA	NA	NA	NA
LSD_5%_, µg/mL	0.6706	0.7239	2.160	0.7684	1.3178	1.5442	1.6073

*Note*: Results are expressed as mean ± standard deviation (*n* = 3). Different superscript letters within the same column indicate statistically significant differences among means according to one‐way ANOVA followed by the LSD test at *p* < 0.05. LSD_5_% values are expressed in µg/mL and represent the least significant difference at the 5% probability level.

Abbreviations: A_0.5_, concentration providing 0.5 absorbance in reducing‐power assays; IC_50_, concentration required to inhibit 50% of radical activity; NA, not applicable; NT, not tested.

The extract displayed marked radical scavenging potential in both DPPH and ABTS assays, with IC_50_ values of 34.20 ± 0.67 and 33.12 ± 0.52 µg/mL, respectively. These values underscore the extract's electron‐donating capacity and aptitude to neutralize lipophilic (DPPH) and hydrophilic (ABTS) radicals. Notably, these results are consistent with the high abundance of polyhydroxylated flavonoids and phenolic acids revealed by LC‐MS, particularly luteolin, apigenin‐7‐O‐glucoside, and rosmarinic acid, compounds extensively documented for their hydrogen atom and electron transfer properties [82, 83].

Although less potent than synthetic standards like BHA or ascorbic acid, the extract's performance is noteworthy, given the complexity and synergism of natural polyphenolic matrices. The IC_50_ values evaluated through DPPH and ABTS fall within the range reported for other *Salvia* species, such as *S. fruticosa* and *S. officinalis* (1.98–50.8 µg/mL) [1, 84], indicating comparable radical scavenging potential.

Compared to previous reports on *S. balansae* collected from the Mostaganem region, clear chemotypic and functional differences emerge. For instance, Mahdjoub et al. [11] reported higher IC_50_ values for DPPH (545.03 µg/mL) and ABTS (328.95 µg/mL), suggesting lower scavenging capacity relative to the current Aurès‐derived extract. Mokhtar et al. [4] likewise observed moderate antioxidant effects in their methanolic extract, with IC_50_ values of 242.7 ± 7.44 µg/mL (DPPH) and 124.1 ± 9.70 µg/mL (ABTS). Bendrihem et al. [14], working with samples from the same geographic origin as this study (Aurès), reported significantly high radical scavenging capacity, particularly for the methanolic flower extract (DPPH IC_50_ = 22.53 ± 0.33 µg/mL and ABTS IC_50_ = 33.69 ± 0.86 µg/mL), affirming the superior antioxidant performance of Aurès populations.

Studies using aqueous extracts from Mostaganem, such as those by Mekki et al. [12] and Souidi et al. [13], confirmed the antioxidant potential via DPPH and FRAP assays, albeit at higher IC_50_ thresholds (DPPH = 62.90–87.50 µg/mL), reflecting both solvent‐specific and geographical variations.

The ability of *Salvia balansae* methanolic extract to bind transition metal ions was evaluated through an iron‐chelating activity assay using ferrozine, a well‐established colorimetric chelator that forms a stable magenta complex with Fe^2+^. In this competitive system, antioxidants capable of sequestering Fe^2+^ ions inhibit the ferrozine‐Fe^2+^ complex formation, thus providing an indirect measure of chelation efficiency [85].

The extract exhibited moderate chelating activity (IC_50_ = 78.19 ± 1.79 µg/mL), attributable to the structural features of its major polyphenolic constituents. Luteolin, the most abundant compound identified, retains all prerequisites for effective Fe^2+^ coordination, the catechol B‐ring, the 5‐OH/4‐keto site, and the C_2_=C_3_ conjugation, making it the principal contributor to this activity [86, 87]. Rosmarinic acid further contributes through its two ortho‐dihydroxyl units, consistent with its reported antioxidant and metal‐chelating properties [88]. The contribution of quercitrin is structurally attenuated by glycosylation at the 3‐OH position, which blocks the primary chelation site, limiting its role in the observed moderate activity [86, 89]. The coexistence of multiple structurally diverse ligands may enhance chelation through synergistic interactions, underscoring the extract's functional relevance in redox modulation. Although the observed activity was lower than that of EDTA, a potent synthetic chelator, it remains significant in the context of natural product matrices.

Compared to prior reports on *S. balansae*, our extract's chelating efficacy aligns closely with the methanolic leaf and stem extracts evaluated by Bendrihem et al. [14], which ranged between 44.45 and 90.24 µg/mL. This reinforces the relevance of methanolic aerial tissues in capturing metal‐binding phytochemicals. In contrast, Mahdjoub et al. [11] reported significantly weaker chelating activity in their hydroethanolic leaf extract (IC_50_ = 689.40 ± 0.86 µg/mL), likely reflecting differences in solvent polarity and organ specificity. These discrepancies emphasize the importance of extraction conditions and plant parts in modulating metal chelation efficiency.

Beyond *S. balansae*, various *Salvia* species exhibit chelation‐mediated antioxidant activity. For instance, *S. officinalis* demonstrates protective effects against heavy metal toxicity, including uranium, through both radical‐scavenging and metal‐binding mechanisms [90]. In a comparative study, Mervić et al. [78] reported Fe^2+^‐chelating IC_50_ values ranging from 163.02 µg/mL in *S. sclarea* to over 1500 µg/mL in *S. officinalis*, *S. fruticosa*, and *S. verticillata*, suggesting that chelating capacity is not solely dependent on total phenolic or rosmarinic acid content.

These findings collectively emphasize the genus's potential in metal detoxification and antioxidant defense, although interspecific variability suggests that chelating efficacy is shaped by both phytochemical composition and ecological adaptation.

Reducing capacity, which reflects the electron transfer potential of antioxidants, was interrogated using three independent methods, CUPRAC, O‐phenanthroline, and SNPAC. The extract demonstrated moderate reducing power in the CUPRAC assay (A_0.5_ = 52.18 ± 0.62 µg/mL), which is particularly responsive to flavonoids and conjugated polyphenols capable of reducing Cu^2+^ to Cu^+^ [91]. This is corroborated by the presence of luteolin and quercitrin in the phytochemical profile, compounds consistently showing high CUPRAC activity in literature [92, 93].

In the O‐phenanthroline assay, the MESB exhibited a moderate iron‐reducing capacity with an A_0.5_ value of 93.36 ± 0.52 µg/mL. This assay is particularly responsive to compounds capable of reducing Fe^3+^ to Fe^2+^, forming an orange–red complex with o‐phenanthroline [94]. Although the extract's iron‐reducing capacity was less pronounced than in the CUPRAC assay, the result remains notable, considering the complexity of natural matrices and the variation in redox potential among metal ions. The observed activity likely reflects the contribution of ortho‐dihydroxylated polyphenols such as luteolin and rosmarinic acid, which are capable of transferring electrons to Fe^3+^ centers and stabilizing the resulting Fe^2+^ through coordination [89, 95, 96].

Comparative data from *S. balansae* studies are limited for this assay; however, our extract demonstrated a considerably lower iron‐reducing capacity than various extracts reported by Bendrihem et al., which ranged from 17.71 ± 0.80 µg/mL (methanolic flower extract) to 60.13 ± 0.89 µg/mL (ethanolic stem extract). Similarly, methanolic extracts of *Salvia aegyptiaca* and *S. verbenaca* also demonstrated higher iron‐reducing efficiency, with A_0.5_ values of 18.71 and 27.03 µg/mL, respectively [96]. These findings suggest that the iron‐reducing potential of *S. balansae* is strongly influenced by extraction conditions, plant part, and species‐specific phytochemical composition. Nevertheless, the extract's measurable capacity to reduce ferric ions underscores its relevance in redox‐active antioxidant defense.

The SNPAC assay assesses the ability of phytochemical extracts to reduce silver ions (Ag^+^) into elemental silver (Ag^0^), a process that results in the formation of silver nanoparticles detectable via their characteristic surface plasmon resonance [30, 97]. In this study, the methanolic extract of *Salvia balansae* demonstrated moderate reducing activity, with an A_0.5_ value of 118.81 ± 0.99 µg/mL. The reduction of Ag^+^ to Ag^0^ by polyphenolic constituents indicates the presence of effective electron‐donating groups, such as the conjugated hydroxyl and carbonyl systems found in flavonoids and phenolic acids [98]. Compounds like luteolin and apigenin, both present in the extract, are known for their redox activity and potential to stabilize nanoparticles via surface interactions and π–π stacking [99]. This electron transfer ability not only confirms the antioxidant nature of the extract but also suggests its potential utility in green nanotechnology, where phytochemicals act simultaneously as reducing and capping agents [97].

The MESB was further evaluated for its capacity to neutralize superoxide anion radicals (O_2_^−^•) using the alkaline DMSO assay, yielding a relatively high IC_50_ value of 690.75 ± 0.43 µg/mL. This suggests a limited scavenging ability compared to standard antioxidants like BHT (IC_50_ = 23.73 ± 1.11 µg/mL). Although superoxide is moderately reactive, it plays a pivotal role in oxidative stress by acting as a precursor to highly reactive and toxic species such as hydroxyl radicals (•OH), peroxynitrite (ONOO^−^) and singlet oxygen (^1^O_2_), which can initiate lipid peroxidation and inflict damage on proteins, nucleic acids, and cell membranes. contributing to aging, inflammation, and neurodegenerative disorders [100].

Although the LC‐MS profile of *S. balansae* revealed the presence of known antioxidant compounds such as luteolin, apigenin‐7‐O‐glucoside, quercetin, and rosmarinic acid, their activity against superoxide radicals may be constrained by glycosylation, steric hindrance, or reduced electron density at reactive sites. Flavonoids like quercitrin and luteolin are recognized for their potential to scavenge O_2_•^−^ through electron donation at hydroxyl groups in the B‐ring. However, glycosidic substitution at position 3, as present in quercitrin, often reduces this activity compared to the free aglycone quercetin [100, 101, 102. The results suggest that while the extract possesses polyphenols with known antioxidant properties, their reactivity toward superoxide is modest, highlighting the selectivity of radical‐scavenging responses and the need for multiassay evaluation to capture the full antioxidant profile of complex plant matrices.

Despite its modest efficacy, the activity of *S. balansae* is within the spectrum observed for other *Salvia* species. For instance, *S. aegyptiaca* and *S. verbenaca* methanolic extracts exhibited IC_50_ values of 129.39 and 136.5 µg/mL, respectively [96], while the essential oil of *S. plebeia* achieved an IC_50_ of 37.3 µg/mL [103]. More recently, *Salvia rosmarinus* was reported to exhibit potent superoxide scavenging activity with IC_50_ ranging from 34.12 to 127.51 µg/mL, with its ethyl acetate fraction showing IC_50_ values nearly equivalent to BHT and vitamin C, while its crude extract also showed remarkable efficacy [104]. These comparative data place *S. balansae* at the lower end of the activity scale, which may be attributed to differences in phytochemical concentration, polarity, and molecular conformation impacting radical accessibility.

The multiassay antioxidant profiling reveals that MESB operates through multiple antioxidant pathways, including radical quenching, metal ion sequestration, and redox potential restoration. These effects are mechanistically linked to the extract's rich phytochemical content, particularly its high levels of flavones (luteolin, apigenin), flavonols (quercetin, rutin), phenolic acids (rosmarinic, caffeic, syringic), and diterpenoids. The synergistic interplay among these metabolites likely amplifies overall antioxidant performance.

In conclusion, the antioxidant performance of *S. balansae* methanolic extract, supported by robust phytochemical evidence, affirms its relevance as a natural multifunctional antioxidant.

### 3.6 Anti‐Inflammatory Activity

The anti‐inflammatory potential of MESB extract was evaluated using the bovine serum albumin (BSA) denaturation assay, a widely accepted model for identifying agents capable of mitigating inflammation‐induced protein destabilization. The extract demonstrated dose‐dependent inhibition of protein denaturation, with a maximal inhibition of 94.69 ± 0.27% at 2500 µg/mL and an IC_50_ value of 1290 ± 0.02 µg/mL (Table 5). In contrast, the reference drug diclofenac sodium exhibited significantly stronger activity, achieving an IC_50_ of 67.97 ± 0.016 µg/mL.

**TABLE 5:** Protein denaturation inhibition percentage of MESB.

C, µg/mL	*S. balansae* extract	Diclofenac sodium
62.5	4.32 ± 0.28	46.64 ± 2.17
125	5.48 ± 0.21	66.69 ± 1.23
250	10.29 ± 0.97	92.29 ± 0.19
375	17.99 ± 0.74	99.68 ± 0.28
500	20.50 ± 0.76	99.98 ± 0.04
1500	57.66 ± 0.27	—
2500	94.69 ± 0.27	—
IC_50,_ µg/mL	1290 ± 0.02^a^	67.97 ± 0.016^b^
LSD_5%_ (IC_50_ only)	0.0364	

*Note*: Values are expressed as mean ± SD (*n* = 3). Different superscript letters within the same row indicate significant differences between the IC_50_ values of *S. balansae* extract and the reference drug according to one‐way ANOVA followed by the LSD test (*p* < 0.05). LSD_5_% denotes the least significant difference at the 5% probability level and applies only to the comparison of IC_50_ values.

Abbreviation: IC_50_, concentration required to inhibit 50% of protein denaturation.

Although the efficacy of *S. balansae* extract was markedly lower than that of diclofenac, its capacity to attenuate protein denaturation at high concentrations remains noteworthy. The observed disparity in potency between *S. balansae* extract and diclofenac sodium is consistent with expectations when comparing complex phytochemical mixtures to purified pharmaceutical compounds with defined molecular structures and specific mechanisms of action. Although requiring higher concentrations, the extract's capacity to inhibit >90% of protein denaturation is pharmacologically relevant and suggests the presence of biologically active constituents with noteworthy anti‐inflammatory properties.

The anti‐inflammatory activity observed in MESB may be attributed, at least in part, to its rich phytochemical composition, particularly its abundance of flavonoids such as luteolin and apigenin. These compounds are well‐documented for their anti‐inflammatory efficacy and may act through multiple mechanisms. One primary pathway involves their antioxidant capacity, which enables the scavenging of reactive oxygen and nitrogen species (ROS and RNS), thereby mitigating oxidative stress‐induced protein denaturation and subsequent inflammatory cascade [105, 106]. Additionally, luteolin and apigenin have been shown to downregulate the expression of key proinflammatory enzymes, such as cyclooxygenase (COX) and lipoxygenase (LOX), leading to reduced synthesis of prostaglandins and leukotrienes, critical mediators of inflammation [75].

These flavonoids also interfere with intracellular signaling by inhibiting the activation and nuclear translocation of nuclear factor kappa B (NF‐κB), a transcription factor that regulates genes encoding cytokines, chemokines, and adhesion molecules involved in the inflammatory response [107]. Collectively, these molecular actions offer a plausible explanation for the extract's in vitro efficacy and underscore its promise as a natural source of anti‐inflammatory agents.

Comparatively, other *Salvia* species have demonstrated variable in vitro anti‐inflammatory efficacy, largely influenced by differences in phytochemical composition, extraction methods, and the specific assay employed. For instance, *Salvia verbenaca* polyphenolic extracts exhibited moderate inhibitory activity against heat‐induced protein denaturation, with an IC_50_ value of 133.21 µg/mL, along with notable membrane‐stabilizing properties [108]. In contrast, *Salvia officinalis* extracts showed relatively weaker activity in the same assay, with an IC_50_ of 5.0 mg/mL, despite demonstrating significant cyclooxygenase‐1 (COX‐1) inhibition (IC_50_ = 19.1 µg/mL), indicating an alternative anti‐inflammatory mechanism via suppression of prostaglandin biosynthesis [109]. Furthermore, *Salvia ballotiflora* extracts, particularly those enriched in diterpenoids from chloroform fractions, displayed potent dose‐dependent anti‐inflammatory effects in both acute and chronic in vivo models, supported by significant downregulation of nitric oxide and proinflammatory cytokines such as TNF‐α, IL‐1β, and IL‐6 [110].

These findings position *Salvia balansae* as a potential source of anti‐inflammatory agents, warranting further investigation in biological models to substantiate its therapeutic relevance.

### 3.7 Cholinesterase Inhibitory Activity

The enzymatic inhibitory potential of the MESB (methanolic extract of *Salvia balansae*) was assessed against acetylcholinesterase (AChE) and butyrylcholinesterase (BChE), two clinically relevant enzymes targeted in the symptomatic treatment of Alzheimer's disease. Inhibition of these enzymes enhances cholinergic transmission, alleviating cognitive deficits associated with neurodegeneration. The results are summarized in Table 6.

**TABLE 6:** Comparative in vitro evaluation of anticholinesterase and antidiabetic activities of MESB.

Extracts	Anticholinesterase activity		Antidiabetic activity
	AChE assay IC_50_, µg/mL	BChE assay IC_50_, µg/mL	α‐AmylaseInhibitory assay IC_50_, µg/mL
*S. balansae* extract	>200^a^	>200^a^	379.8 ± 1.09^b^
Galantamine	5.29 ± 0.37^b^	41.09 ± 1.04^b^	—
Acarbose	—	—	3650 ± 1.86^a^
LSD_5%_	0.3809	1.1633	2.7728

*Note*: Values are expressed as IC_50_ (µg/mL, mean ± SD, *n* = 3). Galantamine and acarbose were used as reference inhibitors for cholinesterase and α‐amylase assays, respectively. >200” indicates that 50% inhibition was not achieved at the maximum tested concentration of 200 µg/mL. Different superscript letters within the same column indicate statistically significant differences among means according to one‐way ANOVA followed by the least significant difference (LSD) test at *p* < 0.05. LSD_5_% values represent the minimum difference required for significance at the 5% probability level.

Abbreviations: AChE, acetylcholinesterase; BChE, butyrylcholinesterase.

In our study, the MESB exhibited negligible in vitro inhibitory activity against both AChE and BChE, with IC_50_ values exceeding 200 µg/mL, under the tested range of concentrations and assay conditions. In contrast, the reference compound galantamine showed potent inhibition of AChE (IC_50_ = 5.29 ± 0.37 µg/mL) and BChE (IC_50_ = 41.09 ± 1.04 µg/mL). These results are in agreement with previous work by Mokhtar et al. [4], who reported a lack of significant cholinesterase inhibition by *S. balansae* methanol extracts (IC_50_ > 200 µg/mL). Additionally, similar findings were noted in *S. glutinosa*, *S. nemorosa*, and *S. sclarea*, which failed to reach 50% inhibition in standard assays [78].

In contrast, several *Salvia* species display markedly stronger cholinesterase inhibition, highlighting the genus's chemical and pharmacological diversity. Among 12 Iranian species, the essential oil of *S. mirzayanii* exhibited the highest activity, inhibiting AChE and BChE by 72.68% and 40.6%, respectively, at 500 µg/mL, with most species showing greater BChE than AChE inhibition [111]. Methanolic aerial‐part extracts of *S. officinalis*, *S. cryptantha*, *S. virgata*, and *S. tchihatcheffii* inhibited AChE by 43.96%, 32.72%, 12.1%, and 5.9%, respectively, at 200 µg/mL [112]. Notably, the n‐hexane fraction of *S. haematodes* showed selective AChE inhibition (IC_50_ = 22.9 µg/mL) and notable BChE inhibition (IC_50_ = 30.9 µg/mL) [113].

The limited cholinesterase inhibitory activity of *S. balansae* reflects the complex interplay between phytochemical composition and enzyme‐binding potential in *Salvia* species. Comparative studies demonstrate that essential oils can exhibit substantially stronger cholinesterase inhibition than corresponding crude extracts when the dominant bioactive metabolites are low‐polarity terpenoids. For instance, in *S. macrosiphon*, the essential oil displayed notable AChE inhibition, whereas the total extract showed no detectable inhibitory effect, a difference attributed to its high content of lipophilic monoterpenes such as linalool, β‐cedrene, and β‐elemene [114]. In contrast, the methanolic extract of *S. balansae* (MESB) is dominated by hydrophilic polyphenols, including rosmarinic acid, salvianolic acids, luteolin, and apigenin derivatives.

While members of these classes such as rosmarinic acid, luteolin, and apigenin derivatives have been reported to inhibit AChE and BChE to varying degrees, their potency is generally moderate compared to standard cholinesterase inhibitors [115, 116]. Rosmarinic acid, for instance, shows measurable but relatively low activity, and combinations of phenolic acids with flavonoids frequently lack synergy or even display reduced inhibition relative to their individual effects, likely due to steric constraints within the enzyme's active site [117]. This activity profile aligns with structural considerations, as AChE possesses a narrow aromatic gorge formed by residues such as Phe295 and Phe297, favoring compact, planar ligands with a basic nitrogen center, whereas in BChE these positions are replaced by smaller aliphatic residues (Leu286 and Val288), resulting in a broader acyl‐binding pocket that accommodates bulkier, hydrophobic ligands [118].

Weak activity under the tested concentrations and assay conditions may also reflect low levels of active compounds in the crude extract, antagonistic interactions among constituents [119, 120], or degradation during incubation. Moreover, assay parameters such as enzyme origin and pH may not fully replicate the human cholinergic environment, potentially underestimating physiological relevance [121, 122].

Notably, modest in vitro inhibition does not preclude neuroprotective potential. El‐Sawi et al. [123] demonstrated that methanolic fractions of *S. splendens*, despite moderate in vitro AChE inhibition by rosmarinic acid and caffeic acid, significantly ameliorated Alzheimer's‐likepathology in an AlCl_3_‐induced rat model. The butanol‐soluble fraction reduced AChE activity in brain tissue by 68.66%, improved neurotransmitter levels, and preserved hippocampal histoarchitecture. These results indicate that cognitive benefits may arise from multifactorial mechanisms, such as antioxidant and anti‐inflammatory effects, modulation of neurotrophic signaling, and restoration of redox balance, rather than direct cholinesterase blockade.

Taken together, although *S. balansae* does not exhibit potent direct inhibition of AChE or BChE, its phytochemical profile, coupled with evidence from related *Salvia* species, supports further exploration of its indirect neuroprotective potential. The absence of high‐potency cholinesterase inhibition may also imply a lower risk of adverse effects associated with strong inhibitors, enhancing its suitability for long‐term cognitive health strategies.

### 3.8 In Vitro α‐Amylase Inhibitory Activity

Inhibition of α‐amylase, a key digestive enzyme, is a validated strategy for moderating postprandial hyperglycemia in type 2 diabetes [124]. Accordingly, the α‐amylase inhibitory activity of MESB was evaluated to assess its potential role in postprandial glucose regulation. The extract exhibited low in vitro activity, with an IC_50_ of 379.8 ± 1.09 µg/mL (Table 6). Low inhibitory activity for *S. balansae* has also been reported by Mokhtar et al. [4], who found IC_50_ values exceeding 400 µg/mL for α‐amylase and α‐glucosidase in aqueous and petroleum ether extracts, placing both studies in a consistent low‐activity range.

However, in vivo studies revealed contrasting results. Mekki et al. [12] observed moderate α‐amylase inhibition in a high‐fat diet model (IC_50_ = 2.20 ± 0.06 mg/mL), and Souidi et al. [13] demonstrated improved glycemic control and metabolic markers in streptozotocin‐induced diabetic rats treated with *S. balansae*. This discrepancy suggests that indirect effects such as antioxidant activity, anti‐inflammatory action, and improvements in insulin signaling may underpin its in vivo efficacy. Such discrepancies between in vitro and in vivo results are well documented in medicinal plant research, as in vivo efficacy may arise from factors absent in isolated enzyme systems, including metabolic activation, attenuation of oxidative stress and inflammation, enhancement of insulin sensitivity, and tissue‐level interactions [125, 126]. Similar patterns have been reported for alkaloid‐rich *Salvia chudaei* extracts, which showed modest in vitro enzyme inhibition but marked in vivo glucose‐lowering effects [127]. Thus, low in vitro α‐amylase inhibition does not preclude meaningful antidiabetic potential, as in vivo conditions can reveal additional mechanisms contributing to efficacy.

Within the broader *Salvia* genus, α‐amylase and α‐glucosidase inhibitory capacities span a wide spectrum, from very weak to highly potent, depending on species‐specific chemistry. For example, Mamache et al. [96] reported α‐amylase IC_50_ values of 86 and 101 µg/mL for methanolic extracts of *S. aegyptiaca* and *S. verbenaca*, respectively, while Mervić et al. [78] documented much weaker α‐glucosidase inhibition for Mediterranean *Salvia* ethanolic extracts (IC_50_ = 4451–5291 µg/mL). Conversely, fractionation can markedly enhance potency, Mahdi et al. [128] found that the ethyl acetate fraction of *S. officinalis* exhibited strong inhibition of both α‐amylase (IC_50_ = 46.52 µg/mL) and α‐glucosidase (IC_50_ = 104.58 µg/mL), attributable to its high phenolic and flavonoid content. This interspecific variability underscores the influence of qualitative phytochemical composition, extraction solvent, and plant part on enzyme inhibition profiles [129].

The comparatively low α‐amylase inhibitory activity of MESB can be attributed to its specific phytochemical composition and the structural properties of its major constituents. LC–MS profiling revealed high levels of glycosylated flavonoids such as quercitrin, rutin, and apigenin‐7‐O‐glucoside, alongside abundant luteolin, rosmarinic acid, syringic acid, and caffeic acid. While aglycones such as luteolin and apigenin are recognized as potent α‐amylase and α‐glucosidase inhibitors through favorable hydrogen‐bonding and hydrophobic interactions within the catalytic site [124, 130], glycosylation generally reduces α‐amylase inhibitory activity by increasing molecular size and steric hindrance, thereby limiting access of the flavonoid scaffold to the enzyme's active site and reducing binding affinity [124, 131, 132]. Because the predominant flavonoids identified in MESB were largely glycosylated (rutin, quercitrin, and apigenin‐7‐O‐glucoside), this structural feature likely contributed to the relatively modest α‐amylase inhibitory activity observed. Additionally, although phenolic acids such as rosmarinic and caffeic acid can interact near the catalytic domain, they generally exhibit weaker α‐amylase inhibition than phenolic diterpenes like carnosic acid or rosmanol, which are abundant in more active *Salvia* species [124, 133]. Collectively, these qualitative phytochemical determinants, rather than total phenolic content alone, likely underlie the low in vitro α‐amylase inhibitory activity of MESB, despite containing compounds with proven in vivo antidiabetic potential.

Finally, weak α‐amylase inhibition in vitro does not rule out significant antidiabetic potential, as plant extracts may lower glycemia through complementary mechanisms such as α‐glucosidase inhibition, modulation of glucose transporters, and mitigation of oxidative stress and inflammation. Accordingly, despite MESB's modest α‐amylase inhibition under assay conditions, its overall efficacy may remain pharmacologically significant within a multifactorial therapeutic context.

### 3.9 Acute Oral Toxicity of *Salvia Balansae*

Acute toxicity testing, typically performed in rodents following a single‐dose administration, constitutes a critical component of preclinical safety assessment. It establishes an initial safety margin, informs dose selection for subsequent studies, and identifies early target‐organ effects, thereby providing a scientific basis for advancing pharmacological evaluation and potential clinical translation [134].

**FIGURE 2:**
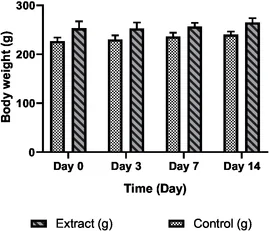
Body weight changes in rats during the 14‐day observation period following acute oral administration of MESB. Values are expressed as mean ± SD. Body weights remained comparable between the control and MESB‐treated groups throughout the study, with no statistically significant differences detected (*p* > 0.05).

In the present study, following OECD Guideline 423, a single oral dose of MESB was administered to adult rats. No mortality or overt signs of toxicity were recorded over the 14‐day observation period. Animals maintained regular locomotor activity, grooming, feeding, and hydration patterns. Body weights remained comparable between the control and MESB‐treated groups throughout the 14‐day observation period (Figure 2), with no statistically significant differences detected (*p* > 0.05), indicating preserved growth and metabolic status. Absolute and relative liver and kidney weights also showed no significant differences between control and treated rats (Table 7). As these parameters are sensitive indicators of organ‐specific toxicity, their stability suggests preserved functional mass [135]. Gross examination revealed no lesions or abnormal coloration, further supporting the absence of hepatic or renal impairment at the tested acute dose. The LD_50_ of MESB was, therefore, estimated to be over 2000 mg/kg.

**TABLE 7:** Absolute and relative liver and kidney weights in control and MESB‐treated rats following acute oral administration.

Parameter	Control	MESB‐treated
Liver weight, g	5.52 ± 0.18	5.57 ± 0.60
Liver weight (% body weight)	2.43 ± 0.06	2.39 ± 0.22
Right kidney weight, g	0.702 ± 0.04	0.684 ± 0.066
Left kidney weight, g	0.655 ± 0.05	0.651 ± 0.060
Total kidney weight, g	1.357 ± 0.085	1.335 ± 0.118
Total kidney weight (% body weight)	0.60 ± 0.03	0.58 ± 0.04

*Note*: Values are expressed as mean ± SD (*n* = 6 per group). Liver and kidney weights are presented as absolute values (g) and relative to body weight (%). No statistically significant differences were observed between control and MESB‐treated groups (*p* > 0.05, unpaired *t*‐test).

These findings align with previous reports of high acute tolerance to *S. balansae*. Souidi et al. [13] and Mekki et al. [12] similarly observed no mortality or adverse clinical signs in rats administered aqueous extracts at doses up to 2 g/kg, confirming a wide safety margin for both methanolic and aqueous preparations. Comparable safety profiles have been reported for related *Salvia* species, although dose‐dependent biochemical alterations have occasionally been noted under subchronic exposure [136, 137].

### 3.10 Histopathological and Morphometric Analysis

#### 3.10.1 Liver

**FIGURE 3:**
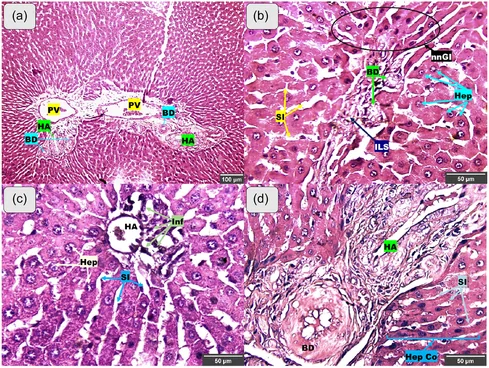
Histological features of rat livers following acute oral administration of *Salvia balansae* methanolic extract. (a) Control group (CTR) showing normal lobular architecture with a well‐preserved portal triad comprising the portal vein (PV), hepatic artery (HA), and bile duct (BD). H&E, ×100; scale bar = 50 µm. (b) Extract‐treated group (EXT) displaying focal non‐necrotizing granulomatous inflammation (nnGl) adjacent to bile ducts (BD), with intact hepatocytes (Hep), sinusoids (SI), and interlobular septa (ILS). H&E, ×100; scale bar = 50 µm. (c) Extract‐treated group (EXT) demonstrating mild portal mononuclear cell infiltration (Inf) surrounding the hepatic artery (HA), with preserved hepatocytes (Hep) and sinusoids (SI). H&E, ×40; scale bar = 50 µm. (d) Extract‐treated group (EXT) showing preserved hepatocyte cords (HepCo), bile duct (BD), hepatic artery (HA), and sinusoids (SI). H&E, ×40; scale bar = 100 µm. Abbreviations: BD, bile duct; HA, hepatic artery; Hep, hepatocytes; HepCo, hepatocyte cords; ILS, interlobular septa; Inf, mononuclear cell infiltration; nnGl, non‐necrotizing granulomatous inflammation; PV, portal vein; SI, sinusoids.

The histological analysis of livers from both control and extract‐treated rats appeared largely unremarkable, with smooth capsules and a uniform reddish‐brown parenchyma. In control animals, hematoxylin–eosin–stained sections demonstrated classic lobular architecture: portal triads comprising a patent portal vein (PV), hepatic artery (HA), and bile duct (BD) were well defined, and hepatic cords (HepCo) radiated regularly from central veins into narrow sinusoids (SI), without any evidence of inflammation, steatosis, or fibrosis (Figure 3, panel a).

In contrast, livers from extract‐treated rats exhibited focal, non‐necrotizing granulomatous inflammation (nnGl). These granulomas consisted of discrete aggregates of epithelioid macrophages within interlobular septa (ILS) adjacent to bile ducts (Figure 3, panel b). Granulomas lacked central necrosis and were rimmed by sparse lymphocytes. Additionally, portal tracts displayed moderate mononuclear cell infiltrates (Inf) of lymphocytes and occasional plasma cells encircling hepatic arteries and bile ducts (Figure 3, panel c). No neutrophilic infiltrates or ductular proliferation were observed.

Despite these inflammatory changes, the overall parenchymal and vascular integrity remained intact. Hepatocyte plates retained their polygonal shape and uniform arrangement, and sinusoids remained patent without congestion or distortion (Figure 3, panel d). Vascular lumina and bile ducts showed no endothelial damage or hyperplasia, and no ballooning degeneration, necrotic foci, or steatotic vacuoles were detected.

Quantitative morphometric analysis corroborated these qualitative observations (Table 8). Hepatocyte cross‐sectional area was 328 ± 22 µm^2^ in treated rats versus 330 ± 20 µm^2^ in controls (*p* = 0.37), and nuclear area remained unchanged (52 ± 5 vs. 52 ± 4 µm^2^, *p* = 0.45). The nuclear‐to‐cytoplasmic ratio likewise showed no difference (0.16 ± 0.02 vs. 0.165 ± 0.015, *p* = 0.48). Vascular indices, including sinusoidal width (6.1 ± 0.4 vs. 6.0 ± 0.5 µm, *p* = 0.29) and central vein diameter (49 ± 3 vs. 50 ± 5 µm, *p* = 0.41), remained stable. Indices of hepatocellular injury such as necrotic area fraction (0.0% vs. 0.1 ± 0.1%, *p* = 1.00) and apoptotic hepatocytes (0 vs. 0.5 ± 0.5 cells/HPF, *p* = 1.00) were essentially absent. Steatosis was negligible (<0.5% vs. 0.5 ± 0.5%, *p* = 1.00), collagen deposition remained low (1.2 ± 0.3% vs. 1.0 ± 1.0%, *p* = 0.54), and inflammatory cell density was comparable (4 ± 2 vs. 5 ± 2 cells/mm^2^, *p* = 0.68).

**TABLE 8:** Morphometric analysis of rat livers following acute oral administration of *S. balansae* extract (mean ± SD, *n* = 6).

Parameter	Control group	Treated group	*p*‐value^a^
Hepatocyte cross‐sectional area	330 ± 20 µm^2^	328 ± 22 µm^2^	0.37
Nuclear area	52 ± 4 µm^2^	52 ± 5 µm^2^	0.45
N/C ratio	0.165 ± 0.015	0.16 ± 0.02	0.48
Sinusoidal width (Zone 3)	6.0 ± 0.5 µm	6.1 ± 0.4 µm	0.29
Central vein diameter	50 ± 5 µm	49 ± 3 µm	0.41
Necrotic area fraction	0.1 ± 0.1%	0.0 ± 0.0%	1.00
Apoptotic hepatocytes	0.5 ± 0.5 cells/HPF	0 ± 0 cells/HPF	1.00
Steatosis (vacuolar area fraction)	0.5 ± 0.5%	<0.5%	1.00
Collagen proportionate area^b^	1.0 ± 1.0%	1.2 ± 0.3%	0.54
Inflammatory cell density^c^	5 ± 2 cells/mm^2^	4 ± 2 cells/mm^2^	0.68

a Unpaired *t*‐test comparing control and treated groups.

b Sirius Red–stained sections.

c Portal + lobular mononuclear cells on H&E.

Quantitative comparison between control and extract‐treated livers revealed no statistically significant differences across all assessed parameters (all *p* > 0.05). Hepatocyte cross‐sectional area (330 ± 20 vs. 328 ± 22 µm^2^) and nuclear area (52 ± 4 vs. 52 ± 5 µm^2^), as well as the nuclear‐to‐cytoplasmic ratio, remained stable, indicating preserved cellular morphology. Vascular patency was maintained, with sinusoidal width and central vein diameter exhibiting minimal variation between groups. Markers of hepatocellular injury (necrotic area fraction and apoptotic hepatocyte count) were essentially zero in both groups, confirming the absence of cytotoxic damage. Lipid accumulation was negligible (vacuolar area fraction < 0.5%), and collagen deposition remained low, demonstrating no fibrotic remodeling. Finally, inflammatory cell density did not differ significantly, reflecting only the mild, focal mononuclear infiltrates seen qualitatively. Collectively, these data support a lack of hepatotoxicity following administration of the plant extract, with all key morphometric indices preserved.

From a toxicological perspective, the presence of focal granulomas and mild portal mononuclear infiltrates suggests a localized, low‐grade immune response, potentially reflecting hepatic processing of phytochemicals or immune activation by plant‐derived antigens [138]. The histological changes observed in our study, focal granulomas and mild portal mononuclear infiltrates without necrosis or steatosis, contrast sharply with the severe hepatotoxicity reported in *Salvia reflexa*. Outbreaks in cattle consuming hay contaminated with *S. reflexa* have been linked to diterpenes such as salviarin and rhyacophiline, which induced acute centrilobular‐to‐panlobular necrosis, hepatocellular swelling, and fatalities within 24–48 h [139, 140]. In contrast, other *Salvia* species, including *S. officinalis*, *S. bucharica*, *S. plebeia*, and *S. miltiorrhiza*, have repeatedly demonstrated hepatoprotective properties in experimental models, mitigating toxin‐induced injury via antioxidant and anti‐inflammatory mechanisms [141, 142, 143, 144]. This dichotomy underscores the chemical diversity of the genus, where diterpenes can act as potent hepatotoxins in some species, while phenolic compounds such as rosmarinic acid confer protection in others [145].

Within this spectrum, *S. balansae* appears to differ markedly from the hepatotoxic profile reported for *S. reflexa*. Although focal non‐necrotizing granulomas and mild portal mononuclear infiltrates were observed in treated animals, these findings occurred in the absence of hepatocellular necrosis, steatosis, vascular alterations, or significant changes in morphometric parameters. The lesions therefore suggest a mild localized inflammatory response rather than overt hepatocellular injury under the conditions of this acute exposure study.

Nevertheless, given the 14‐day duration of the experiment, the biological significance and long‐term evolution of these inflammatory changes cannot be fully determined. Accordingly, while the present results indicate no evidence of acute hepatotoxicity at 2000 mg/kg, additional repeated‐dose toxicity studies following OECD Guidelines 407 (28‐day) and 408 (90‐day) are warranted to establish the chronic safety profile and confirm the hepatic safety margin of the extract.

#### 3.10.2 Kidney

**FIGURE 4:**
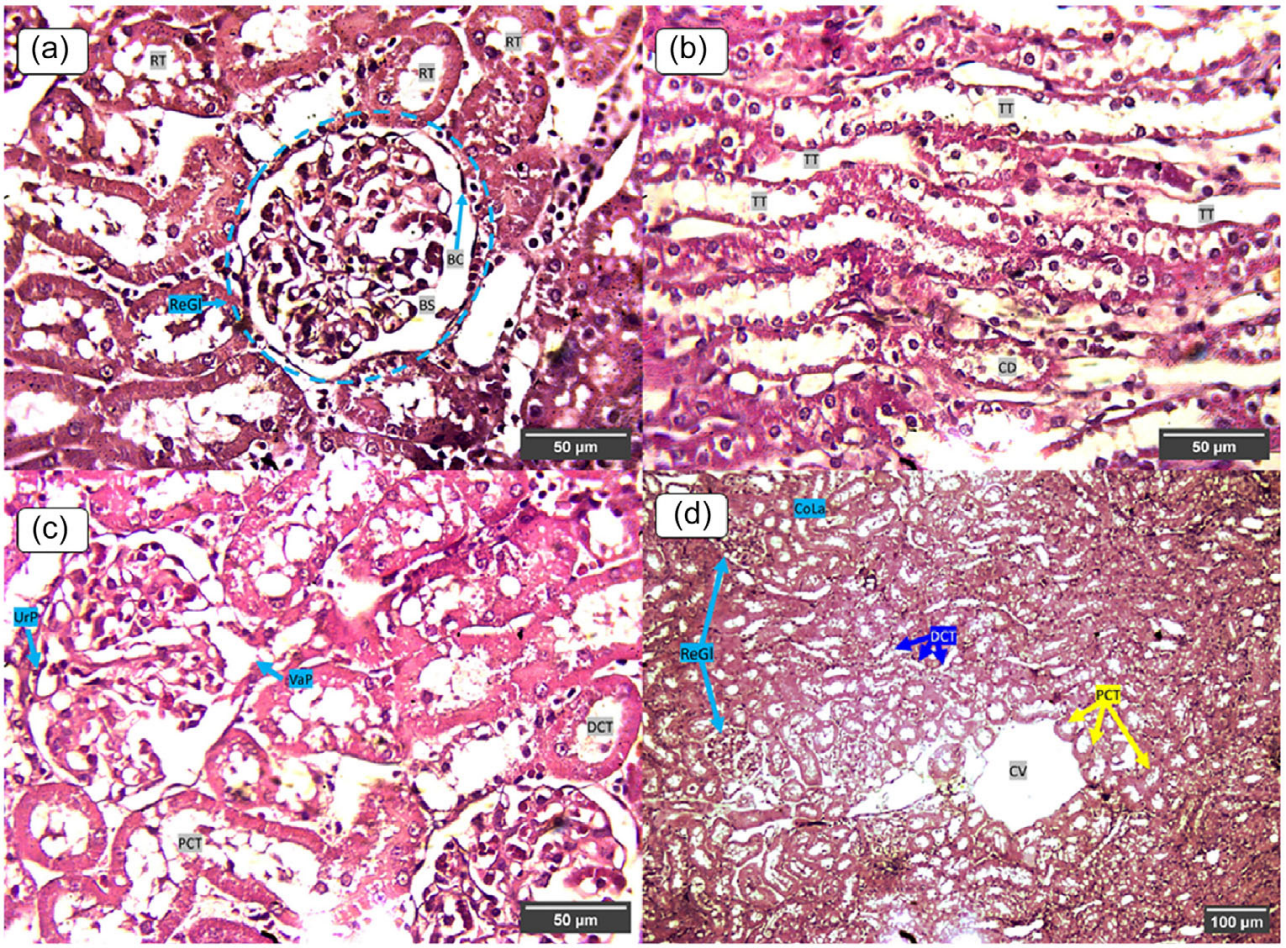
Histological morphology of rat kidneys in control and extract‐treated groups. (a) Normal kidney (CTR): intact renal glomerulus (ReCo) with well‐defined Bowman's space (BS) and capsule (BC), and preserved renal tubules (RT). H&E, ×100; scale bar = 50 µm. (b) Extract‐treated kidney (EXT): normal cortical and medullary structures with thin tubules of Henle's loop (TT) and collecting ducts (CD). H&E, ×100; scale bar = 50 µm. (c) Extract‐treated kidney (EXT): urinary pole (UrP), proximal convoluted tubule (PCT), distal convoluted tubule (DCT), and vascular pole (VaS) preserved without pathological alterations. H&E, ×100; scale bar = 50 µm. (d) Extract‐treated kidney (EXT): cortical labyrinth (CoLa) with intact glomeruli (ReCo), proximal (PCT) and distal (DCT) tubules, and central vein (CV). H&E, ×40; scale bar = 100 µm.

On light microscopy, renal cortical sections from control animals exhibited standard architecture. Renal glomeruli (ReCo) were round to ovoid, with well‐defined Bowman's capsules (BC) and Bowman's spaces (BS) surrounding unremarkable capillary tufts (Figure 4, panel a). Proximal convoluted tubules (PCT) displayed cuboidal epithelium with a prominent brush border, while distal convoluted tubules (DCT) featured a clearer lumen and lower epithelial height. Collecting ducts (Cd) and thin limbs of Henle's loop (tt) in the medulla were similarly normal, and no hyaline casts or apoptotic profiles were observed.

Kidneys from extract‐treated rats retained overall histoarchitecture, with glomeruli and tubular segments indistinguishable from controls (Figure 4, panel b). Bowman's capsule integrity was preserved, and PCT and DCT epithelia showed no cytoplasmic vacuolation or nuclear pyknosis. Interstitial tissue remained scant, and there was no evidence of inflammatory infiltrates, interstitial fibrosis, or tubular necrosis. However, rare hyaline casts were noted within some distal tubules.

Quantitative morphometry confirmed these observations (Table 9). Mean glomerular tuft area in treated rats (11,850 ± 900 µm^2^) did not differ significantly from controls (12,000 ± 800 µm^2^; *p* = 0.68). Bowman's space width, PCT epithelial thickness, and DCT epithelial thickness likewise showed no statistical alteration (*p* ≥ 0.55). Tubular lumen area fraction (58 ± 6%) and interstitial area fraction (8.2 ± 1.3%) were comparable to control values (60 ± 5% and 8.0 ± 1.2%, respectively; *p* = 0.43 and 0.65). Apoptotic tubular cells remained rare (0.8 ± 0.9 vs. 0.5 ± 0.7 cells/mm^2^; *p* = 0.48). The only parameter reaching statistical significance was hyaline casts, present at 1.5 ± 1.2 casts/mm^2^ in treated kidneys versus none in controls (*p* = 0.04), indicating a mild, transient proteinaceous tubular precipitation without overt tubular injury.

**TABLE 9:** Morphometric parameters in control and extract‐treated rat kidneys.

Parameter	Control group	Treated group	*p*‐value^a^
Glomerular tuft area, µm^2^	12,000 ± 800	11,850 ± 900	0.68
Bowman's space width, µm	4.5 ± 0.3	4.6 ± 0.4	0.55
PCT epithelial thickness, µm	13.0 ± 1.0	13.2 ± 1.1	0.72
DCT epithelial thickness, µm	10.0 ± 0.8	10.1 ± 0.9	0.81
Tubular lumen area fraction, %	60 ± 5	58 ± 6	0.43
Interstitial area fraction, %	8.0 ± 1.2	8.2 ± 1.3	0.65
Apoptotic tubular cells, cells/mm^2^	0.5 ± 0.7	0.8 ± 0.9	0.48
Hyaline casts, casts/mm^2^	0 ± 0	1.5 ± 1.2*	0.04*

a Unpaired *t*‐test; *p < 0.05; mean ± SD; *n* = 6 per group.

The preservation of normal glomerular morphology, tubular epithelial thickness, luminal patency, and interstitial volume attests to maintained renal structure following extract administration. The slight but significant increase in hyaline casts suggests minimal tubular protein precipitation without concomitant apoptosis or inflammatory damage, arguing against overt structural nephrotoxicity at the tested dose. Hyaline casts, primarily composed of Tamm‐Horsfall protein, can appear in both physiological and pathological states [146]. Their isolated occurrence without structural or inflammatory alterations is more consistent with a transient, subclinical event than with toxic injury, though functional confirmation is lacking.

Our findings align with a broader body of research on *Salvia* species, which more often demonstrate nephroprotective than nephrotoxic effects. Salvianolic acid C attenuates cisplatin‐induced nephrotoxicity by reducing oxidative stress, inflammation, and apoptosis while preserving renal histology [147]. Salvianolic acid A exerts antifibrotic and anti‐inflammatory effects in nephrectomized rat kidneys through inhibition of NF‐κB and MAPK signaling [148]. Likewise, *Salvia miltiorrhiza* injections ameliorated lead‐induced renal injury by decreasing apoptosis and lipid peroxidation [149], while chia (*S. hispanica*) seed extract restored tubular and glomerular integrity in Ehrlich ascites carcinoma–bearing mice [150]. Reports of nephrotoxicity within the genus remain scarce and context‐dependent. A subchronic study of purple sage extract described increased kidney weights and indices, suggesting caution with prolonged administration [151], whereas salvinorin A exposure produced no renal histological damage even at high doses [152].

The statistically significant increase in hyaline casts (1.5 ± 1.2 casts/mm^2^ vs. 0 in controls; *p* = 0.04), although isolated, warrants cautious interpretation. The preservation of glomerular morphology, tubular epithelial integrity, and the absence of apoptosis or interstitial inflammation argue against overt structural nephrotoxicity. However, the functional significance of this finding cannot be fully assessed because serum creatinine, blood urea nitrogen (BUN), and urinary protein measurements were not performed. This limitation should be considered when interpreting the renal safety profile of the extract, and future repeated‐dose studies should incorporate biochemical markers of renal function to determine whether the observed hyaline casts represent a transient adaptive response or an early indicator of functional stress. These findings align with reports of other *Salvia* species with high renal safety margins [147, 149, 150], though longer‐term studies with biochemical markers remain necessary to fully establish its nephrotoxic profile.

### 3.11 In Vivo Anti‐Inflammatory Activity of MESB

Evaluation of anti‐inflammatory activity in vivo provides a more physiologically relevant assessment than in vitro assays, as it integrates vascular, cellular, and tissue‐level responses that are not captured by protein stabilization models such as BSA denaturation [153]. The croton oil–induced ear edema model is particularly well established as a rapid and sensitive method for detecting topical anti‐inflammatory effects, since it reproduces the complex interplay of vascular permeability, epidermal hyperplasia, and leukocyte infiltration characteristic of acute skin inflammation [154].

The anti‐inflammatory effect of *S. balansae* extract was confirmed through two complementary endpoints: ear thickness and ear punch biopsy weight. Ear thickness reflects acute edema and vascular permeability, providing a dynamic measure of early mediator activity, whereas biopsy weight quantifies cumulative tissue swelling and exudate accumulation at the endpoint, making it a more precise histological correlate [155, 156].

Our results demonstrate that topical application of *Salvia balansae* extract exerts a clear, dose‐dependent anti‐inflammatory effect in the croton oil‐induced ear edema model. The croton oil challenge increased ear thickness by 27.8% compared with negative controls (CTL), confirming the robustness of the model. Treatment with indomethacin (IND) reduced swelling by 90.9%, validating the assay, while the extract produced 51.5% and 57.6% inhibition at doses of 10 and 20 mg/ear, respectively. When evaluated by ear biopsy weight, the extract demonstrated even stronger efficacy, achieving 63.4% and 75.4% inhibition, with the higher dose producing a response nearly equivalent to indomethacin (78.2%). as shown in Tables 10 and 11. This pattern suggests that the extract may exert a stronger effect on tissue‐associated inflammatory processes, such as cellular infiltration and exudate accumulation, beyond superficial vascular permeability changes.

**TABLE 10:** Effect of MESB on croton oil‐induced ear edema in rats: changes in ear thickness and percentage inhibition relative to control.

Group	Diameter difference, cm ± SD	% Inhibition^a^
Negative control (CTL)	0.055 ± 0.016	—
Indomethacin (IND)	0.005 ± 0.005	90.9^***^
Extract 10 mg/ear	0.027 ± 0.014	51.5^**^
Extract 20 mg/ear	0.023 ± 0.012	57.6^**^

a Values are mean ± SD (*n* = 6). % Inhibition calculated relative to CTL. **p < 0.01, ***p < 0.001 vs. CTL.

**TABLE 11:** Effect of *Salvia balansae* extract on croton oil‐induced ear edema in rats: changes in ear weight and percentage inhibition relative to control.

Group	Weight difference, mg ± SD	% Inhibition^a^
Negative control (CTL)	3.55 ± 4.40	—
Indomethacin (IND, 0.1 mg/ear)	0.78 ± 1.08	78.2^***^
Extract 10 mg/ear	1.30 ± 3.72	63.4^**^
Extract 20 mg/ear	0.88 ± 0.54	75.4^***^

a Values are mean ± SD (*n* = 6). % Inhibition calculated relative to CTL. **p < 0.01, ***p < 0.001 vs. CTL.

**FIGURE 5:**
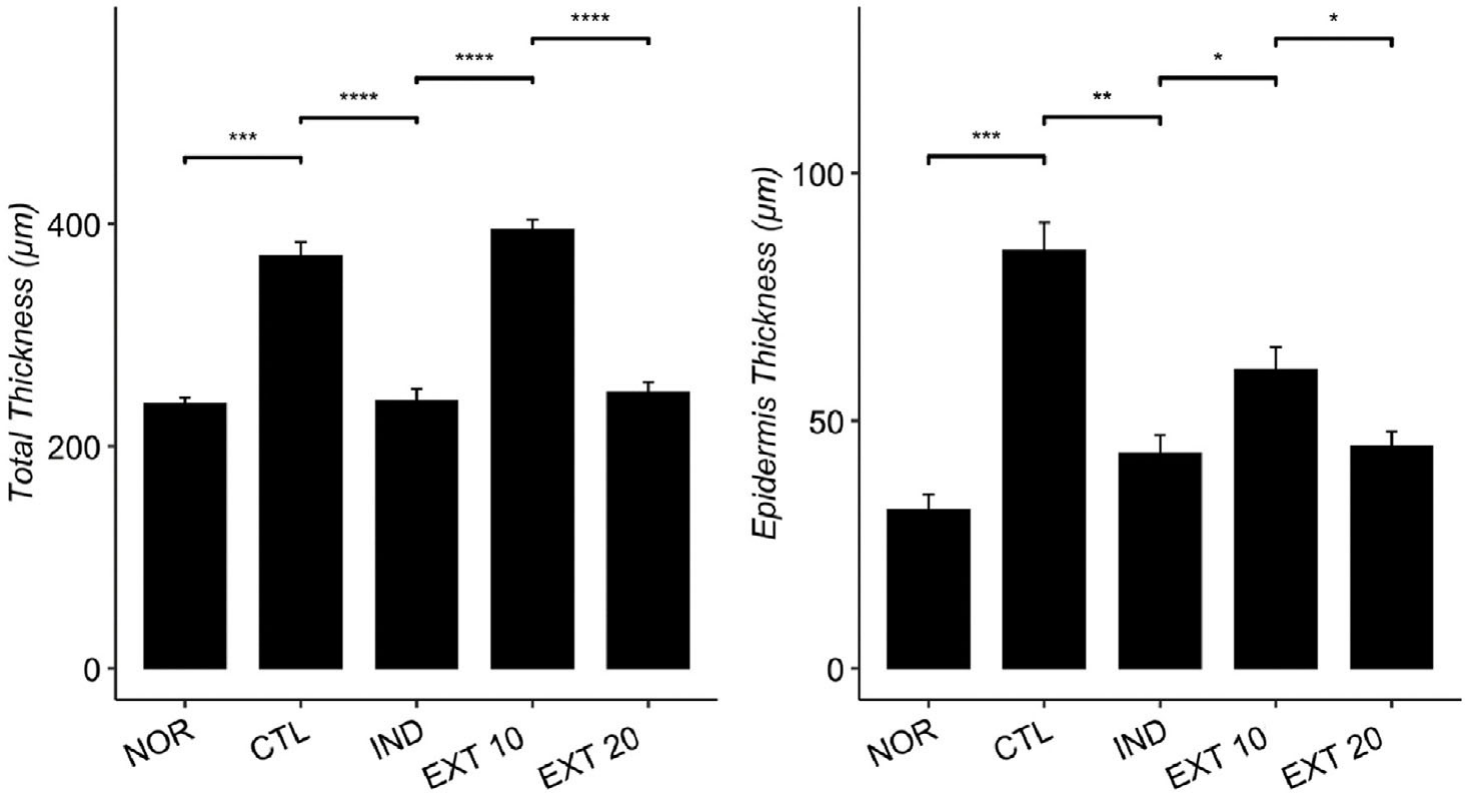
Quantitative analysis of total ear thickness and epidermal thickness (mean ± SEM) across experimental groups; adjacent‐pair significance (Student's *t*‐test) indicated. *p < 0.05; **p < 0.01; ***p < 0.001; ****p < 0.0001. NOR, normal control; CTL, vehicle control; IND, indomethacin; EXT 10, extract (10 mg/ear); EXT 20, extract (20 mg/ear).

Histomorphometric measurement of epidermal thickness (Figure 5) mirrored the edema data and corroborated these findings. Croton oil induced a 164.5% increase in epidermal thickness, accompanied by spongiosis, inflammatory infiltration, and vascular congestion. Treatment with the extract significantly reversed these pathological alterations, with the higher dose reducing epidermal hyperplasia by 47.7%, approaching the level of indomethacin (48.8%). The congruence between macroscopic, histological, and biopsy weight measurements reinforces the biological relevance of the extract's anti‐inflammatory properties.

The mechanistic implications indicate that croton oil–induced inflammation is strongly driven by neutrophil‐derived mediators. Proteomic and immunohistochemical studies have confirmed early upregulation of MPO and MMP‐9 as well as the presence of MMP‐8 in inflamed ear tissue [154, 157]. Chronic exposure models further show time‐dependent increases in MMP‐9 activity and collagen degradation [158, 159]. The ability of *S. balansae* extract to suppress epidermal thickening and attenuate biopsy weight gain suggests interference with these pathways, possibly through modulation of cytokines such as TNF‐α, IL‐1β, and IL‐6, which are known to mediate both early vascular permeability and sustained cellular recruitment [160, 161].

Furthermore, the pharmacological activity of *S. balansae* is consistent with previous reports on other *Salvia* species, many of which are rich in bioactive phenolic acids (e.g., rosmarinic, caffeic), flavonoids (e.g., luteolin, apigenin), and pentacyclic triterpenoids (e.g., ursolic, oleanolic, betulinic acids), which demonstrated potent inhibition of croton oil–induced inflammation [156, 162, 163, 164]. These compounds have been shown to modulate key inflammatory pathways, including cyclooxygenase (COX) and lipoxygenase (LOX) inhibition, suppression of NF‐κB activation, and downregulation of proinflammatory cytokines such as TNF‐α, IL‐1β, and IL‐6. The strong efficacy observed in vivo, therefore, likely reflects the synergistic action of these phytochemical classes [15, 75]**.** Importantly, the activity level demonstrated here places *S. balansae* among the more pharmacologically active members of the *Salvia* genus, positioning it as a promising but previously unexplored candidate for phytotherapeutic development.

**FIGURE 6:**
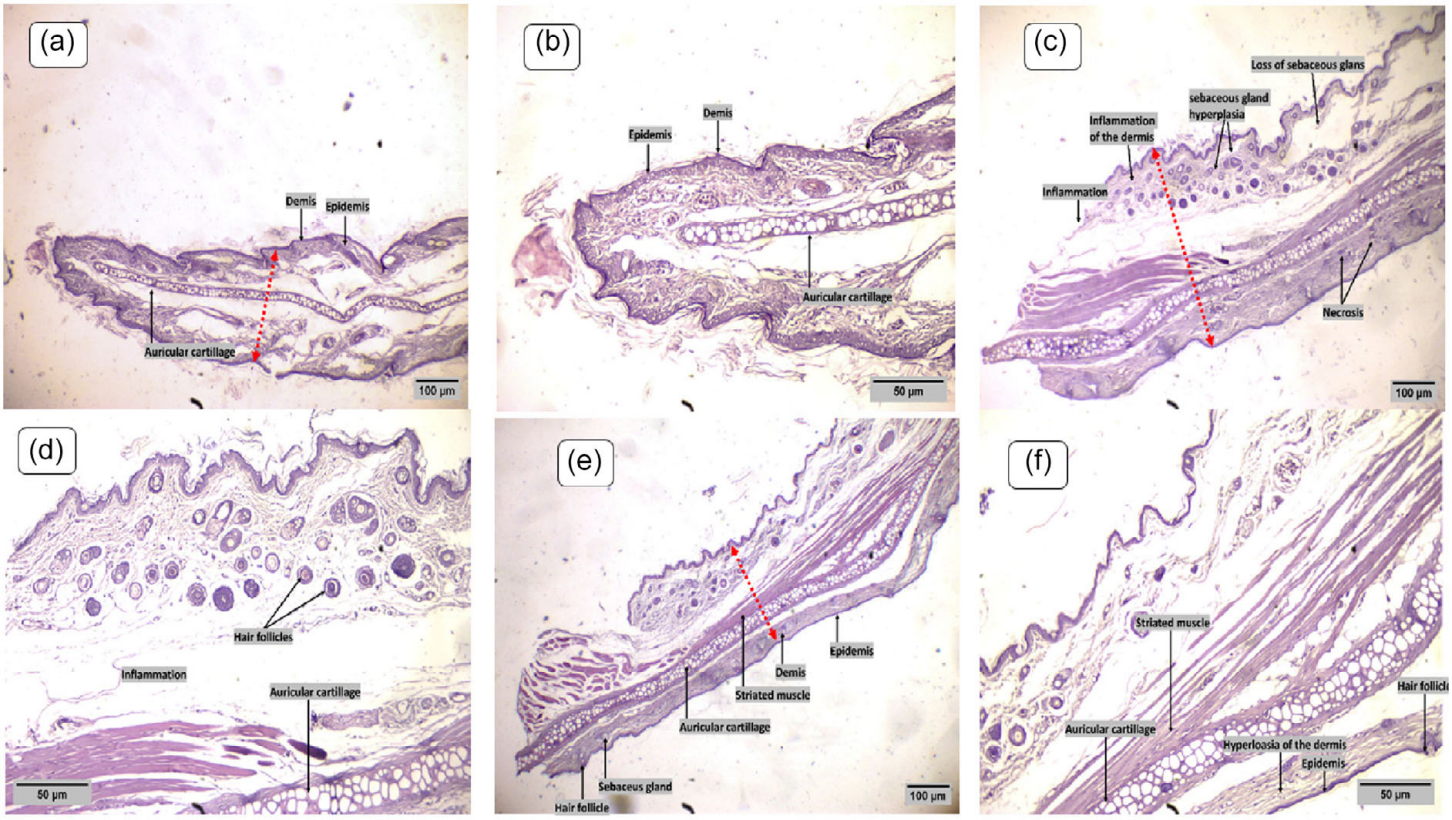
Histopathological changes in Wistar rat ears following croton oil–induced inflammation and treatments (panels a–f). H&E stain. (a) Normal ear architecture, ×40; (b) normal ear architecture, ×100; (c) dermal and epidermal changes 6 h after croton oil–induced inflammation, ×40; (d) croton oil–induced inflammation, ×100; (e) protective effects of indomethacin on croton oil–induced edema, ×40; and (f) indomethacin‐treated ear showing hyperplasia of the dermis, ×100.

**FIGURE 7:**
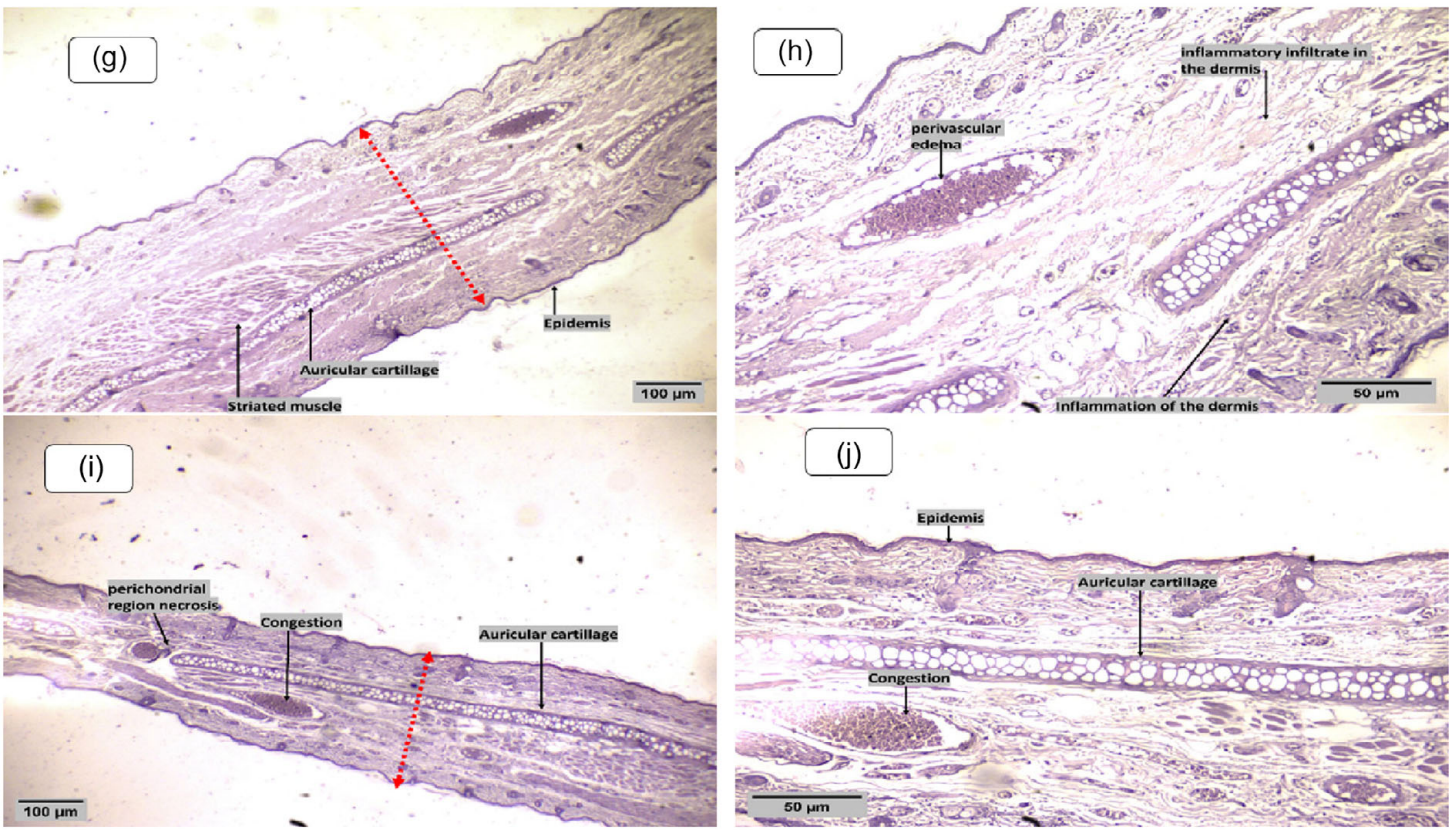
Histopathological changes in Wistar rat ears following treatment with MESB (panels g–j). H&E stain. (g) Histological appearance after treatment with MESB 10 mg/ear, ×40; (h) MESB 10 mg/ear, ×100, showing residual perivascular edema and inflammatory infiltrate in the dermis; (i) histological appearance after treatment with MESB 20 mg/ear, ×40, showing perichondrial region necrosis and congestion; and (j) MESB 20 mg/ear, ×100, showing residual congestion with preserved epidermal and cartilage architecture.

Histopathological examination revealed that in the ears of normal nontreated animals (Figure 6a,b), the epidermis was thin and uniform, the dermis contained sparse fibro‐adipose tissue, and sebaceous glands and hair follicles appeared normal. In the negative control group (Figure 6c,d), marked epidermal hyperplasia, focal spongiosis, and a dense neutrophilic–mononuclear infiltrate were observed in the dermis, accompanied by perivascular edema and occasional areas of keratinocyte necrosis. Indomethacin‐treated ears (Figure 6e,f) showed near‐normal epidermal thickness and a moderate reduction of dermal infiltrate. Application of the plant extract at 10 mg/ear (Figure 7g,h) produced only partial reversal of hyperplasia and a patchy decrease in inflammatory cells, whereas the 20 mg/ear dose (Figure 7i,j) restored epidermal architecture and substantially diminished cellular infiltration, comparable to IND. These histological improvements correlated with the quantitative measures of thickness and support a dose‐dependent anti‐inflammatory effect of the plant extract.

In summary, *Salvia balansae* extract demonstrated significant, dose‐dependent anti‐inflammatory activity in vivo, effectively reducing edema, epidermal hyperplasia, and inflammatory cell infiltration in the croton oil ear model, with efficacy comparable to indomethacin. Its preferential modulation of deeper tissue inflammatory processes highlights its potential as a topical phytotherapeutic candidate for localized skin and soft tissue inflammation. It should be acknowledged, however, that rats exhibit thicker ears and a comparatively less sensitive cutaneous inflammatory response than mice, which may affect absolute edema magnitude; our comparisons nonetheless remain internally consistent, as all experimental and control groups were subjected to identical conditions within the same animal model [165]. The gravimetric endpoint (Table 11) exhibited high inter‐individual variability, with SD values exceeding group means in the CTL and EXT‐10 groups; ear thickness (Table 10) is nonetheless considered the primary quantitative endpoint, with biopsy weight serving a corroborative role, supported by independent histological evidence (Figures 5, 6, 7). Nonetheless, the acute nature of this model and the reliance on histological and gravimetric endpoints limit the mechanistic resolution of our findings. Future studies should therefore integrate cytokine profiling, MPO and MMP activity assays, and upstream pathway analyses (NF‐κB, MAPK, Nrf2), alongside long‐term models such as carrageenan paw edema and Freund's adjuvant arthritis, to better define the therapeutic window and translational relevance of this extract.

### 3.12 Molecular Docking

The NLRP3 (NOD‐like receptor protein 3) inflammasome is a multiprotein complex that plays a crucial role in the innate immune response. When activated, NLRP3 triggers caspase‐1 activation, leading to the processing and secretion of proinflammatory cytokines IL‐1β and IL‐18, thereby initiating inflammatory responses [166]. Dysregulation of NLRP3 activity has been implicated in various inflammatory and autoimmune diseases, including rheumatoid arthritis, inflammatory bowel disease, atherosclerosis, and neurodegenerative disorders [167]. Furthermore, NLRP3 has emerged as a critical player in Alzheimer's disease pathogenesis and type 2 diabetes, with evidence suggesting it potentially links obesity‐associated inflammation and insulin resistance with cognitive impairment [168].

MCC950 is a potent and selective small‐molecule inhibitor of NLRP3 that directly binds the NACHT domain, interacting with the Walker B ATP‐hydrolysis motif to prevent ATP hydrolysis and inflammasome activation [169]. Because its binding mode has subsequently been resolved by both X‐ray crystallography and cryo‐electron microscopy [36, 170], MCC950 is widely used as a reference inhibitor in computational studies investigating potential NLRP3‐targeting compounds. Although MCC950 has shown promising efficacy in preclinical models, concerns regarding hepatotoxicity have limited its clinical translation, increasing interest in safer natural‐product‐derived NLRP3 modulators [171].

**FIGURE 8:**
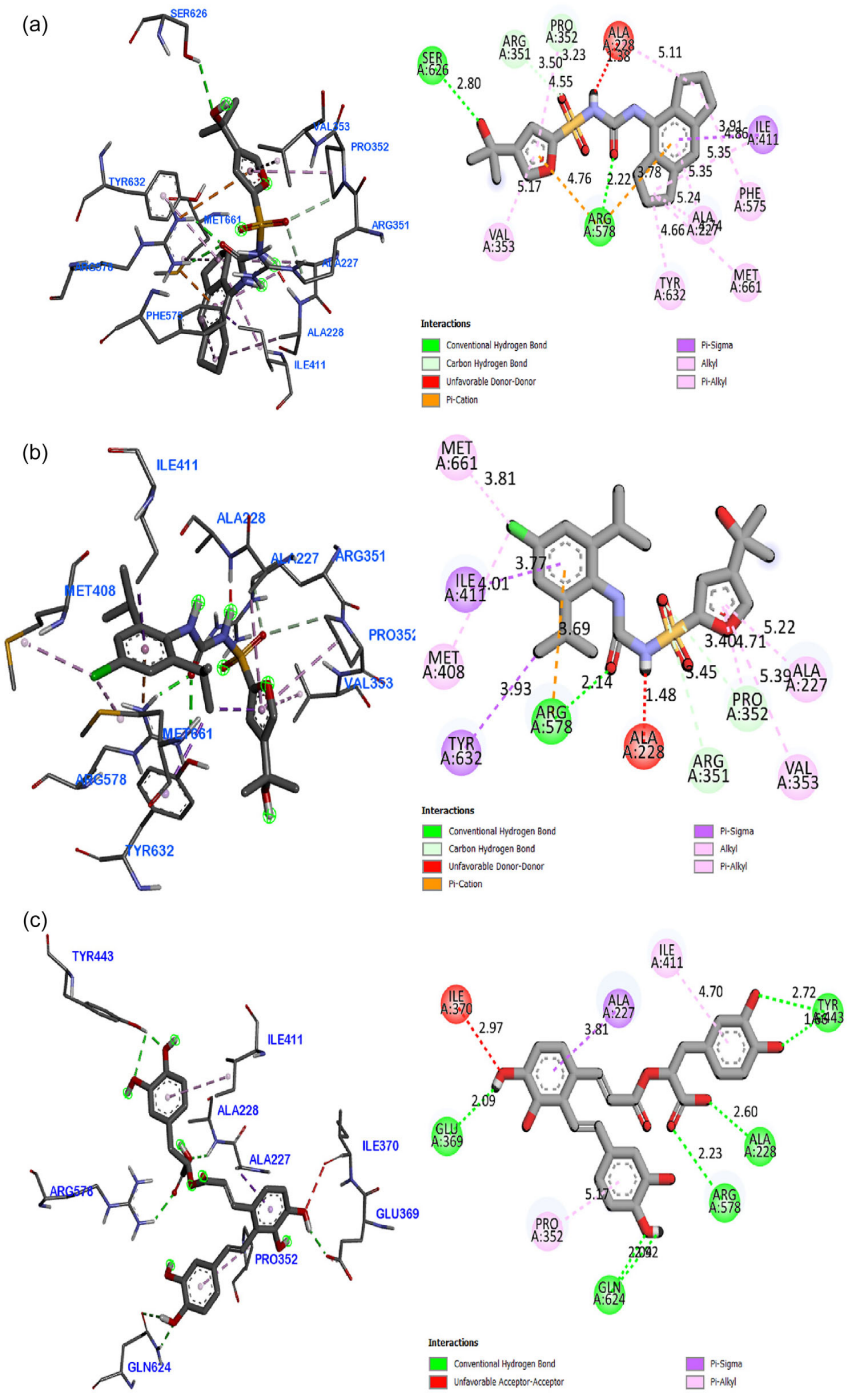
3D/2D binding modes of inhibitors and phytochemicals from *Salvia balansae* docked into the binding pocket of NLRP3 (PDB: 7ALV): (a) MCC950–NLRP3, (b) native ligand RM5–NLRP3, and (c) salvianolic acid–NLRP3.

To assess the anti‐inflammatory potential of NLRP3 inhibition by MESB, molecular docking analysis was conducted on fifteen phytochemicals and two reference compounds, evaluating their binding affinities with the NLRP3 inflammasome target. The results, including binding energy values (kcal/mol), are summarized in Table 12. More negative docking scores indicate stronger binding affinities, suggesting a higher likelihood of effective molecular interactions between the compounds and the target protein. The coligand redocking resulted in acceptable RMSD values, demonstrating the method's accuracy and reliability for predicting potential natural NLRP3 inhibitors. The predicted binding modes and key interacting residues of the reference inhibitor MCC950, the native ligand RM5, and salvianolic acid (the top‐ranked phytochemical) within the NLRP3 binding pocket are shown in Figure 8a–c, respectively.

**TABLE 12:** Docking results of phytocompounds from MESB with the target protein NLRP3 (PDB: 7ALV).

Ligand	Binding energy, kcal/mol	Hydrogen bonding, Å	Hydrophobic bonding, Å	Electrostatic interaction, Å
MCC950 (inhibitor)	−10.4	ARG578 (2.37, 2.07), SER626 (2.80), ARG351 (3.50), PRO352 (3.23)	ALA227 (5.24), ALA228 (5.11), ILE411 (5.35, 3.91), MET661 (4.74), PHE575 (4.86), TYR632 (4.66), PRO352 (4.55), VAL353 (5.17)	ARG578 (3.78, 4.76)
Salvianolic acid B	−9.7	ALA228 (2.60), TYR443 (2.72)	—	—
RM5 (native ligand)	−9.5	TYR443 (1.66)	—	—
Rutin	−9.3	ARG578 (2.23)	—	—
Apigenin‐7‐O‐glucoside	−9.1	GLN624 (2.04)	—	—
Luteolin	−8.7	GLN624 (2.92)	—	—
Quercitrin	−8.5	GLU369 (2.09)	—	—
Chlorogenic acid	−8.5	—	ALA227 (3.81)	—
Acacetin	−8.3	—	ILE411 (4.70)	—
Apigenin	−8.2	—	PRO352 (5.17)	—
Naringenin	−8.2	—	—	—
Epicatechin	−8.1	—	—	—
Rosmarinic acid	−8.0	ARG578 (2.02)	—	—
Caffeic acid	−6.0	ARG578 (2.25)	—	—
Syringic acid	−5.8	ARG351 (3.45)	—	—
O‐coumaric acid	−5.8	PRO352 (3.40)	—	—
Quinic acid	−5.6	—	ILE411 (3.77), TYR632 (3.93), MET408 (4.01), MET661 (3.81), ALA227 (5.22), PRO352 (4.71), VAL353 (5.39)	ARG578 (3.69)

Abbreviation: Å, interaction distance expressed in angstroms, as calculated from the docked complexes using BIOVIA Discovery Studio Visualizer.

The binding energies of the phytochemicals from MESB compared to the reference molecules MCC950 and RM5 ranged from −5.6 to −9.7 kcal/mol (Table 12). Phytocompounds with binding energies equal to or lower than those of the reference inhibitors are considered as potentially bioactive against the target protein.

According to our results, salvianolic acid expressed the highest affinity (−9.7 kcal/mol) against NLRP3, approaching the reference inhibitor MCC950 (−10.4 kcal/mol) and exceeding the native ligand RM5 (−9.5 kcal/mol). By forming hydrogen bond interactions with key residues ALA228 and TYR443 in the active site of the target protein, salvianolic acid emerged as the most promising candidate in the docking analysis and may represent a potential NLRP3 inhibitor. The complex salvianolic acid‐NLRP3 exhibited specific hydrogen bonding patterns that may contribute to favorable binding within the predicted binding pocket.

Rutin and apigenin‐7‐O‐glucoside also demonstrated high binding affinities (−9.3 and −9.1 kcal/mol, respectively), forming hydrogen bonds with ARG578 and GLN624, respectively. These residues appear to be crucial for ligand binding in the NLRP3 active site. These computational findings are supported by experimental evidence demonstrating that flavonoids, including apigenin and structurally related compounds, suppress NLRP3 inflammasome assembly through inhibition of ASC oligomerization and the Syk/Pyk2 signaling pathway in cellular models [172]. The growing pharmacological interest in natural NLRP3 modulators [173] further contextualizes the relevance of the *S. balansae* flavonoid profile as a source of potential inflammasome‐targeting candidates.

Luteolin (−8.7 kcal/mol), quercitrin (−8.5 kcal/mol), and chlorogenic acid (−8.5 kcal/mol) also exhibited favorable binding energies, suggesting their potential as NLRP3 inhibitors.

In contrast, caffeic acid, syringic acid, o‐coumaric acid, and quinic acid displayed the highest binding energies (−6.0 to −5.6 kcal/mol), indicating lower affinities to the NLRP3 active site.

Importantly, the best‐scored phytochemicals (salvianolic acid, rutin, apigenin‐7‐O‐glucoside) interacted with residues also highlighted in the validated MCC950 binding pocket, including ARG578, ARG351, PRO352, and ILE411. This convergence is particularly significant because molecular dynamics simulations by Nascimento et al. [174] identified ALA227, ALA228, PRO352, ILE411, PHE575, and ARG578 as key contributors to the stability of MCC950 and its analog NP3−146 within the NLRP3 binding site. The shared involvement of PRO352, ILE411, and ARG578 suggests that these phytochemicals may occupy a similar binding region and engage interaction networks comparable to those reported for established NLRP3 inhibitors.

This consistency extends to other recent in silico studies. Rocha et al. [171] showed that natural compounds from *Euterpe oleracea* (catechin, apigenin, epicatechin) formed favorable complexes with NLRP3 by engaging MCC950‐associated residues. Sayed et al. [175] reported even lower binding energies (–12.3 kcal/mol) for flavonoids from *Mimusops elengi* at the ADP‐binding site, underscoring the drug‐like potential of natural phenolics. N. Singh et al. [176] and Hayat et al. [177] further confirmed, via molecular dynamics and MM‐PBSA, that hydrogen bonding, van der Waals forces, and electrostatic interactions are the main stabilizing contributors in phytochemical–NLRP3 complexes.

Taken together, the docking results suggest that several *S. balansae* metabolites, particularly salvianolic acid, rutin, and apigenin‐7‐O‐glucoside, may interact favorably with the NLRP3 binding pocket and share binding‐site features reported for known NLRP3 inhibitors. These findings provide a plausible molecular basis for the anti‐inflammatory activity observed experimentally; however, docking predictions alone do not establish target engagement or biological efficacy. Additional computational approaches, such as molecular dynamics simulations and free‐energy calculations, together with experimental validation, are required to confirm the role of NLRP3 inhibition in the pharmacological effects of the extract.

## 4 Conclusion

This work provides the first comprehensive characterization of *Salvia balansae* from the Aurès Mountains, revealing it as a chemically distinctive and pharmacologically valuable species. The methanolic extract of aerial parts was found to be exceptionally rich in flavonoids, phenolic acids, and triterpenoids, with luteolin and rosmarinic acid emerging as major constituents that define its unique chemotype. This phytochemical richness translated into strong and multifaceted antioxidant activity, confirmed across complementary radical‐scavenging, metal‐chelating, and reducing‐power assays, highlighting the synergistic interplay of its polyphenolic constituents. The extract further demonstrated significant anti‐inflammatory effects, both in vitro through inhibition of protein denaturation and in vivo by reducing croton‐oil–induced ear edema and restoring normal histological architecture. Computational docking studies suggested favorable interactions between several major metabolites and the NLRP3 inflammasome, indicating a possible molecular interaction that may contribute to the observed anti‐inflammatory activity; however, these in silico findings should be regarded as hypothesis‐generating and require further validation through molecular dynamics simulations and target‐based experimental studies. Direct inhibitory activity against cholinesterases and α‐amylase was modest, suggesting that potential neuroprotective and antidiabetic effects may arise indirectly through antioxidant and anti‐inflammatory mechanisms. Acute toxicity evaluation revealed no mortality or overt organ damage at 2000 mg/kg, although mild localized hepatic inflammatory changes were observed, and repeated‐dose studies are required to fully establish the long‐term safety profile of the extract. Together, these findings identify *S. balansae* as a promising source of antioxidant and anti‐inflammatory phytochemicals. Its distinctive chemotype and broad bioactivity profile warrant further investigation through fractionation, pathway‐specific assays, and disease‐model studies to clarify its mechanisms of action and support future pharmaceutical and nutraceutical development.

## Funding

The authors have nothing to report. 

## Ethics Statement

All experimental procedures involving animals were conducted in accordance with institutional guidelines for the ethical treatment of animals. Ethical approval for this study was obtained from the University of El Oued, Algeria (reference no. 03/2024).

## Statement of Informed Consent

Not applicable. This study did not involve human participants.

## Conflicts of Interest

The authors declare that they have no known competing financial interests or personal relationships that could have appeared to influence the work reported in this paper.

## Statement of Human and Animal Rights

This study was conducted in accordance with institutional and international guidelines for the care and use of laboratory animals. All procedures were approved by the appropriate ethics committee and complied with the ethical standards concerning animal welfare. No human participants were involved in this study.

## Supporting information

Supplementary Material

## Data Availability

The datasets used and analyzed during the current study are available from the corresponding author on reasonable request.
