# Supplementary material for: Integrative Phytochemical, Antioxidant, and NLRP3‐Linked and Anti‐Inflammatory Evaluation of Salvia balansae (Aurès Chemotype): Insights Into Its Safety and Therapeutic Potential
Source: ChemistryOpen. 2026 Aug 20;15(9):e70280. doi: 10.1002/open.70280 (PMC13494366; doi:10.1002/open.70280)
Supplement: Supplementary file 1 [file OPEN-15-e70280-s001.pdf]

Supplementary Table S1. Limits of Detection (LOD) and Limits

	LOD (mg/L)	LOQ (mg/L)
quinic acid	0.0005	0.0015
Gallic acid	0.0001	0.0003
Protocatechuic acid	0.0002	0.0005
Chlorogenic acid	0.0002	0.0005
caffeic acid	0.0004	0.0012
1,3-di-O-caffeoylquinic acid	0.0017	0.0051
syringic acid	0.0007	0.002
Epicatechin	0.0043	0.0131
trans-Ferulic acid	0.0001	0.0003
Rutin	0.0001	0.0003
Hyperoside (quercetin-3-O-galactoside)	0.0002	0.0007
o-Coumaric acid	0.0001	0.0013
Naringin	0.0014	0.0044
Quercitrin (quercetin-3-O-rhamnoside)	0.0002	0.0005
Apigenin-7-O-glucoside	0.0002	0.0005
Rosmarinic acid	0.0007	0.002
Salvianolic acid	0.0012	0.0035
quercetin	0.0006	0.0019
trans-Cinnamic acid	0.0009	0.0027
Naringenin	0.0002	0.0007
Apigenin	0.0001	0.0003
Luteolin	0.0018	0.0054
Cirsiliol	0.0003	0.0008
Cirsilineol	0.0005	0.0017
Acacetin	0.0002	0.0006

Abbreviations: LOD, limit of detection; LOQ, limit of quantification. Values are expr
